# Differences in Anatomical Structures and Resting-State Brain Networks Between Elite Wrestlers and Handball Athletes

**DOI:** 10.3390/brainsci15030285

**Published:** 2025-03-07

**Authors:** Fatma Sahin Ozarslan, Adil Deniz Duru

**Affiliations:** Faculty of Sports Science, Marmara University, İstanbul 34815, Türkiye; deniz.duru@marmara.edu.tr

**Keywords:** fMRI, handball, wrestling, neuroplasticity, voxel-based morphometry (VBM), sports

## Abstract

Background/Objectives: Advancements in biomedical imaging technologies over the past few decades have made it increasingly possible to measure the long-term effects of exercise on the central nervous system. This study aims to compare the brain morphology and functional connectivity of wrestlers and handball players, exploring sport-specific neural adaptations. Methods: Here, we examined 26 elite male athletes (13 wrestlers and 13 handball players) using anatomical and resting-state functional magnetic resonance imaging (fMRI) measurements. Connectivity maps are derived using the seed-based correlation analysis of resting-state fMRI, while voxel-based morphometry (VBM) is employed to identify anatomical differences. Additionally, the cortical thickness and global volumetric values of the segmented images are examined to determine the distinctions between elite wrestlers and handball players using non-parametric statistical tests. Results: Wrestlers exhibited greater grey matter volume (GMV) in the right middle temporal gyrus, left middle frontal gyrus, and right posterior cingulate gyrus (uncorr., *p* < 0.001). On the other hand, wrestlers showed increased functional connectivity in the left superior temporal gyrus, left parahippocampal gyrus, the left anterior orbital gyrus, and right superior frontal gyrus–medial frontal region (*P*(_FWE_) < 0.05). In addition, wrestlers showed greater cortical thickness in several brain regions. Conclusions: The increased GMV, cortical thickness, and functional connectivity observed in wrestlers highlight the presence of sport-specific neural adaptations. While this research provides valuable insights into the neuroplastic effects of various athletic disciplines, further studies involving additional sports and control groups are needed for a more comprehensive understanding.

## 1. Introduction

Recent studies show that regular long-term and intense physical exercise cause both physiological [1,2] and anatomical changes [3,4,5,6] in the central nervous system. A cross-sectional study highlights that athletes attain elite status through years of intensive training and early specialization in their respective sports [7]. Improvements in motor control, coordination, perception, and decision-making are observed in elite athletes when they are compared with sedentary people or even with novice athletes. These behavioural changes are due to structural and functional adaptations and are described as occupational neuroplasticity, which can be measured using a variety of neuroimaging techniques [8]. 

In handball, which requires a great ability to adapt to the constantly changing conditions imposed by the play of teammates [9], physical and technical skills, such as holding the ball with one or both hands, passing or diving with force, using sufficient speed and great precision through different trajectories, catching a ball thrown with different trajectories and forces, etc., are required [10]. Wrestlers engage in one-on-one combat, where they attempt to grab, hold, and throw their opponent without striking them, using a number of different offensive and defensive actions. The athlete must attack the opponent’s body directly and the attack can be carried out simultaneously [11]. During these attacks, athletes must constantly adapt their behaviour to the behaviour of their opponents [12].

Previous studies have demonstrated that long-term athletic training induces structural and functional plasticity in the brain. For instance, research using resting-state functional magnetic resonance imaging (fMRI) in elite ice-skating athletes shows increased grey matter volume (GMV) in motor-related brain regions, such as the posterior cerebellum, frontal and temporal lobes, and subcortical structures, along with enhanced functional connectivity patterns [13]. A study conducted by Duru and Balcioglu [4] on elite karate athletes demonstrated increased GMV in motor-related and visual processing regions, such as the premotor cortex and temporal and occipital lobes, as well as greater white matter (WM) integrity in subcortical structures, including the caudate nucleus and hypothalamus. Additionally, enhanced functional connectivity has been reported in movement planning and visual perception networks [4]. A multimodal magnetic resonance imaging (MRI) study, which highlighted structural and functional brain differences in endurance runners compared to non-athletes, found that endurance runners exhibit greater GMV and cortical surface area in motor-related regions, such as the precentral gyrus, alongside increased functional connectivity between motor and somatosensory areas. Additionally, endurance running was associated with greater hippocampal volume and enhanced connectivity between the hippocampus and motor-control regions, as well as higher WM integrity in key pathways, including the corpus callosum and cerebellum [14]. Recent research has shown that long-term training in table tennis enhances visuospatial cognitive processing efficiency and strengthens neuroplasticity in the right hemisphere, the dominant region in spatial cognition [15]. Similarly, numerous studies in the literature have investigated brain plasticity in elite athletes with extensive training in various disciplines, such as basketball, artistic gymnastics, taekwondo, and football, by comparing them with control groups [16,17,18,19,20,21]. Together, these studies provide compelling evidence that long-term, high-level athletic training drives structural and functional adaptations in the brain, with sport-specific patterns of neuroplasticity emerging across different disciplines.

While these studies highlight sport-specific neuroplasticity by comparing athletes to non-athlete controls, fewer studies have directly examined structural and functional brain differences between athletes from distinct disciplines. A study that compared professional handball players and ballet dancers to investigate sport-specific brain adaptations found that ballet dancers exhibited decreased fractional anisotropy in WM fibres connecting both hand and foot areas compared to handball players. Additionally, GMV was increased in the hand regions of handball players, whereas ballet dancers showed greater GMV in the foot regions [22]. Schlaffke et al. [23] compared athletes from sports with different metabolic profiles—such as endurance-based and anaerobic disciplines—and the results show distinct patterns of brain adaptations. Voxel-based morphometry (VBM) analyses revealed that both endurance athletes and martial artists exhibit increased GMV in motor-related areas, such as the supplementary motor area and dorsal premotor cortex. However, endurance athletes uniquely displayed greater GMV in the medial temporal lobe, particularly in the hippocampus and parahippocampal gyrus regions previously associated with aerobic exercise and memory function [23]. A study comparing elite endurance (aerobic) and sprint (anaerobic) athletes found that aerobic athletes exhibited greater GMV in the cerebellum and temporal lobe, while anaerobic athletes showed increased GMV in the basal ganglia. Functional analyses further revealed that aerobic athletes had higher spontaneous neural activity and connectivity in motor-related regions, whereas anaerobic athletes exhibited stronger functional activity in the posterior cerebellum, highlighting distinct brain adaptations associated with different training intensities [24]. Given the unique physical and cognitive demands of different sports, further research is needed to explore how various training regimens shape neural adaptations. In this context, comparing handball and wrestling athletes—two disciplines with distinct motor, cognitive, and metabolic demands—can provide valuable insights into sport-specific brain plasticity.

While comparisons of sports with different metabolic profiles (aerobic vs. anaerobic) [23,24] and movement patterns (hand- vs. foot-dominant sports) [22] are available in the literature, to our knowledge, no study has investigated whether individual and team sports are associated with changes in brain morphology. Moreover, brain imaging studies in this area remain limited. For this purpose, in this study, we investigated the brain’s morphological structural features and functional connections network using fMRI analyses of elite athletes in two different sports disciplines: the team sport handball and the individual combat sport wrestling. Based on these aims, below, two hypotheses are proposed, outlining the concept of this study.

(i) Anatomical structures of the brain tissue represented by the segmented T1 images differ between wrestlers and handball athletes.
(ii) Resting-state networks obtained through the analysis of Blood Oxygenation Level-Dependent (BOLD) signal differ between wrestlers and handball athletes.

The outcome of this study will contribute to expanding the level of interdisciplinary knowledge and identifying morphological adaptations caused by long continuous training and technical skills specific to the sport.

## 2. Materials and Methods

### 2.1. Subjects 

This study examined a cohort of 26 elite Turkish male athletes, with a mean age of 23.7 years (SD = 3.2 years). Participants were required to meet the following inclusion criteria: (1) age between 18 and 30 years, (2) male sex to control for potential hormonal influences, and (3) elite athlete status, defined as participation in nationally ranked and international competitions for individual wrestling athletes or competition in the national super league teams for handball players. Exclusion criteria included standard contraindications for MRI (e.g., claustrophobia, the presence of metal implants such as pacemakers or braces). Additional exclusion criteria included (1) a history of neurological or psychiatric disorders, (2) a history of head injuries or chronic diseases, and (3) current or past substance use.

The participants were evenly divided into two groups: 13 wrestlers (mean age = 22.8 years, SD = 3.3 years) and 13 handball players (mean age = 24.5 years, SD = 2.9 years). The training sessions of these athletes were monitored over the course of one week. It was observed that the wrestlers trained for an average of 9 h and 55 min per week, while the handball players trained for an average of 9 h and 37 min per week. The wrestlers had an average of 13.2 years (SD = 3.5 years) of competitive experience in their sport, while the handball players had an average of 13.5 years (SD = 3.8 years) of competitive experience.

Due to the limited number of specialized athletes in the population, obtaining adequate sample sizes for brain imaging studies is more challenging compared to disease studies. This difficulty is particularly evident in neuroimaging research involving elite athletes, where participant recruitment remains a significant constraint.

For instance, in a recent study, Zhang et al. [25] collected resting-state fMRI data from ten volleyball athletes and ten college students, reporting no significant behavioural differences between the groups. However, the athlete group exhibited an increased Amplitude of Low-Frequency Fluctuations and Regional Homogeneity in the visual cortex, along with stronger functional connectivity between the visual cortex and multiple brain regions, compared to novices. Similarly, a study [2] examined skeleton athletes in comparison to healthy controls, with a sample size of only eleven athletes, further highlighting the challenges of recruiting elite sports participants for neuroimaging research.

This study was conducted in accordance with the Helsinki Declaration and received approval from the Marmara University Ethics Committee on 20 September 2022 (document number 09.2022.1038). All participants provided written informed consent.

### 2.2. MRI Protocol and Methods

Athletes who volunteered for this study completed a questionnaire that gathered demographic information and details about their disease status. The volunteers visited the hospital, where the anatomical MRI and resting-state fMRI measurements were performed. The anatomical image of the participant was acquired using a T1-weighted sequence, with a total scan duration of 5 min. MRI data were collected on a 1.5 Tesla Signa Explorer (GE Healthcare, Chicago, IL, USA) scanner equipped with a 16-channel head coil. A high-resolution T1-weighted 3D image was obtained with the following parameters: a field of view (FOV) of 250 × 250 mm, a voxel size of 1.00 × 1.00 × 1.00 mm, and 180 transverse slices. These were reconstructed to 0.98 × 0.98 × 1.00 mm. Following the anatomical scan, the participant was informed about the resting-state fMRI measurement phase. In resting-state fMRI studies, participants are typically instructed to remain still, stay awake, and think of nothing in particular for a duration ranging from 5 to 15 min [26]. In this study, 5 min of resting-state fMRI data were collected in a single session, yielding 104 volume scans with a repetition time (TR) of 3 s.

Resting-state fMRI data were acquired using a single-shot echo-planar imaging (EPI) sequence in the transverse orientation, with the following parameters: a voxel size of 2.00 × 2.00 × 4.00 mm, a TR of 3000 ms, and an echo time (TE) of 30 ms. All images were then converted from DICOM to NIfTI format using the MRIcron software package for further processing.

The analysis of brain structural differences is performed using classical statistical tests and VBM. Prior to analysis, preprocessing steps are implemented. T1 weighted anatomical images are subjected to coregistration and normalization by the use of the diffeomorphic anatomical registration through exponentiated lie algebra (DARTEL). Then, normalized and segmented images are smoothed for the decrement of the spatial noise. The preprocessing of fMRI data was conducted using SPM12, following standard spatial and temporal correction procedures. First, motion correction was applied through realignment using a 6-parameter rigid-body transformation, with resampling at a voxel size of 4 mm and interpolation set to 4th-degree B-spline. Slice timing correction was then performed to account for temporal differences in slice acquisition, using the middle slice as the reference.

Next, structural and functional images were coregistered, using normalized mutual information as the cost function. This was followed by the tissue segmentation of the T1-weighted image into grey matter, white matter, and cerebrospinal fluid (CSF) using SPM’s tissue probability maps. The estimated deformation fields from segmentation were then used for normalization to an MNI space with a voxel size of 3 mm.

Finally, spatial smoothing was applied using an 8 mm full-width at half maximum Gaussian kernel to enhance signal-to-noise ratio and improve statistical sensitivity. All of the preprocessing steps are conducted using the SPM12.7771 toolbox in MATLAB2024b.

**Figure 1:**
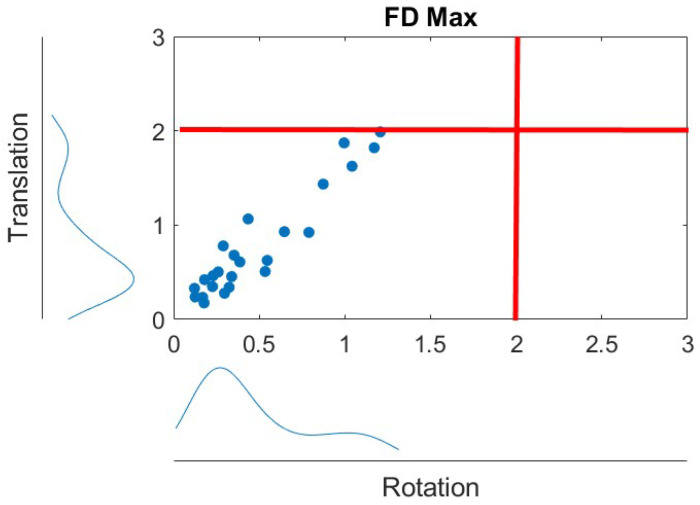
Distributions of maximum framewise displacement in translation and rotation. The red lines indicate the 2 mm and degree thresholds for exclusion criteria. Blue dots stand for the translation and rotation of each subjects data.

During the preprocessing of the resting-state fMRI data, the preprocessing of anatomical MRI data is reconsidered. Anatomical data were segmented into grey matter, WM, and CSF tissue classes using SPM unified segmentation and a normalization algorithm [27,28] with the default IXI-549 tissue probability map template. Functional data were denoised using a standard denoising pipeline [29], including the regression of potential confounding effects characterized by WM time series (5 CompCor noise components), CSF time series (5 CompCor noise components), session effects and their first-order derivatives (2 factors), and linear trends (2 factors) within each functional run. This was followed by bandpass frequency filtering of the BOLD time series [30] between 0.008 Hz and 0.09 Hz. As an alternative to GSR, we applied CompCor [31], which takes the principal components (usually 5 PCs) of WM/CSF regions as regressors in the nuisance regression step. To assess motion-related artefacts, we computed framewise displacement and visualized the distribution of maximum framewise displacement in translation and rotation [32]. The threshold for motion exclusion was set at 2 mm for translation and at 2 degrees for rotation, corresponding to half of the voxel size. Figure 1 illustrates the distribution of maximum framewise displacement in translation and rotation.

**Figure A1:**
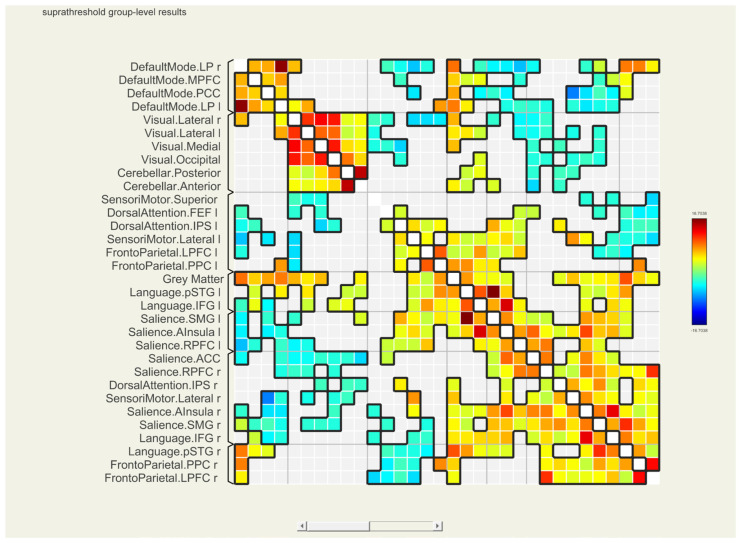
*t* values of the ROI-to-ROI analysis of network level corrected values (*P*(_FWE_) < 0.05, *n* = 26) for all subjects. Red colouring indicates strong positive connectivity, while the blue ones show negatively correlated connectivity. Gray-coloured connections did not achieve the corresponding threshold. Detailed values of the statistics are given in Table A1.

**Figure A2:**
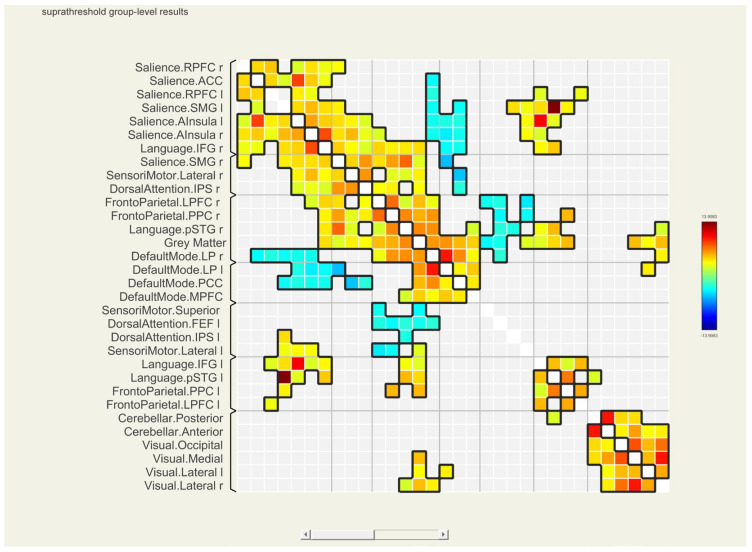
*t* values of the ROI-to-ROI analysis of network level corrected values (*P*(_FWE_) < 0.05, *n* = 13) for Handball players. Red colours indicate the strong positive connectivity while the blue ones show the negative correlated connectivity. Gray-coloured connections did not achieve the corresponding threshold. Detailed values of the statistics are given in Table A2.

**Figure A3:**
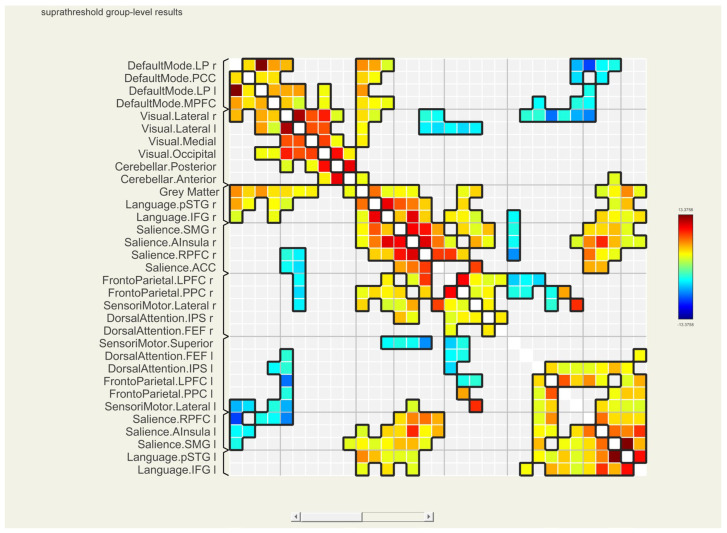
*t* values of the ROI-to-ROI analysis of network level corrected values (*P*(_FWE_) < 0.05, *n* = 13) for Wrestlers. Red colours indicate the strong positive connectivity while the blue ones show the negative correlated connectivity. Gray-coloured connections did not achieve the corresponding threshold. Detailed values of the statistics are given in Table A3.

CONN22 toolbox is adopted to analyze the differences between resting-state functional connectivity patterns of two groups of participants. Seed-based connectivity maps are reconstructed using the central seeds positions of the functional networks in 164 HPC-ICA [33]. The regions of interest (ROIs) used in this study and their corresponding functional networks are detailed in Table 1. These ROIs were selected based on the 164 HPC-ICA parcellation and served as seed locations for functional connectivity analysis. Basically, the Pearson Correlations between BOLD time series of a seed location/ROI (region of interest) and all of the voxels are computed. Then, the Fisher-transformed coefficients are subjected to statistical tests to elucidate the significant connectivity maps. In the Appendix we added t-maps of ROI to ROI Connectivity maps and tables, for all subjects, for Handball players, for Wrestlers (Figure A1, Figure A2 and Figure A3 and Table A1, Table A2 and Table A3 in Appendix A).

### 2.3. Statistical Analysis

#### 2.3.1. Whole-Brain Metric Statistics

For the whole-brain segmented images, Mann–Whitney U tests are performed to check the differences in mean values between two independent groups (wrestlers and handballs player) for the grey matter (GM), white matter (WM), and CSF volumes. The Mann–Whitney U test, also known as the Wilcoxon rank-sum test, is a non-parametric statistical method used to compare two independent groups when the assumption of normality in data distribution is uncertain or not met. Jamovi software version 2.5 (The Jamovi Project 2024, Sydney, NSW, Australia) is used to implement Mann–Whitney U tests. Since averaging values from an area with high spatial resolution results in the removal of hidden information in the data, we performed comparison on voxel-based data and cortical thickness values.

#### 2.3.2. Voxel-Based Morphometry Statistics

We used SPM to perform a voxel-wise two-sample *t*-test to assess group differences while controlling for the confounding variable of total intracranial volume. Since VBM involves thousands of voxel-wise tests, Family-Wise Error (FWE) statistical corrections are applied to control for Type I errors. Clusters of significant differences indicate regions where GMV differs between handball players and wrestlers.

#### 2.3.3. Resting-State Networks Comparison Statistics

Voxel-level hypotheses were evaluated using multivariate parametric statistics with random effects across subjects and sample covariance estimation was performed across multiple measurements. Inferences were performed at the level of individual clusters (groups of contiguous voxels). Cluster-level inferences were based on parametric statistics from Gaussian Random Field theory [29,34]. For the connectivity map results, multiple comparison correction was taken into account for the cluster level and height threshold (*P*(_FWE_) < 0.05).

## 3. Results

### 3.1. Anatomical Differences in Wrestlers and Handball Athletes 

The first objective of this study was to compare the differences in anatomical structures of brain tissue as represented by the segmented T1 images between the wrestlers and the handball players. Thus, in the first step, we checked whether the whole-brain parameters varied between the groups. Among the volumetric parameters, only the GM relative volume parameter was significantly higher for wrestlers than the mean value for the handball players. The descriptive values for the parameters and comparison statistical values are shown in Table 2. Next, we analyze the differences at both the voxel and cluster levels using VBM and cluster thickness parameters.

**Figure 2:**
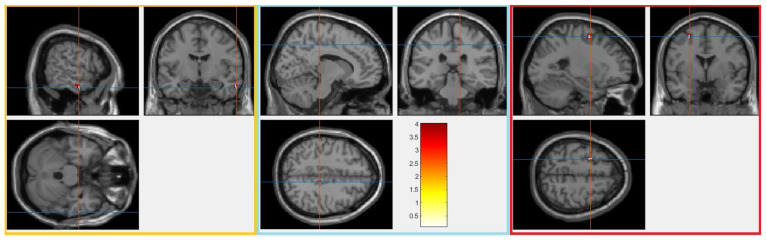
Three clusters of significant grey matter differences (*p* < 0.001, uncorrected) for the contrast wrestlers > handball are highlighted in yellow, light blue, and red boxes. The colour bar represents the corresponding t-statistic values for the contrast. From left to right, the figure displays the regions of middle temporal gyrus (MTG), right posterior cingulate gyrus (PCG), and left middle frontal gyrus (MFG).

When we applied the contrast “wrestler > handball” in VBM analysis, none of the anatomical regions survived after the FWE correction with the 0.05 threshold. Then, we used a more liberal threshold of an uncorrected *p* < 0.001 with an extent threshold of 10 voxels to check for possible differences in GM values between wrestlers and handball players. Finally, the right middle temporal gyrus (MTG), left middle frontal gyrus (MFG), and right posterior cingulate gyrus (PCG) were found to differ, as summarized in Table 3 (Figure 2).

Finally, we performed Mann–Whitney U tests to check the significant differences in the cortical thickness parameters, with a statistical threshold of *p* < 0.05. Several cortical regions were exhibited to have significantly greater cortical thickness values, both in the left and right hemispheres, as summarized in Table 4. In the left hemisphere, the thickness values of the superior occipital sulcus and transverse occipital sulcus (occipital lobe), superior temporal sulcus (STS), and temporal pole (temporal cortex) were found to be higher for the wrestlers. In both hemispheres, the fusiform gyrus was shown to have greater thickness values. As a part of the right limbic gyrus, the subcallosal gyrus thickness value was prominent for wrestlers. Similarly to previous regions, in the right hemisphere, medial orbital sulcus, and lat. orbitofrontal located in the frontal areas were thicker. Among the main portions of the default mode network, the posterior cingulate, and middle–posterior parts of the cingulate gyrus and sulcus were found to have increased cortical thickness values.

Several of the regions identified in our analysis, including the STS, temporal pole, and fusiform gyrus, have been associated with spatial perception, body representation, and multisensory integration—functions closely related to peripersonal space (PPS) processing [35,36,37,38]. While PPS was not the primary focus of this study, the examined brain regions have previously been linked to spatial processing and body representation. Although the observed structural differences do not directly correspond to brain regions traditionally associated with PPS [39], they may still contribute to spatial representation and movement-related functions and potentially have implications how athletes process spatial information in their immediate environment.

### 3.2. Resting-State Functional Connectivity Differences in Wrestlers and Handball Athletes 

**Figure 3:**
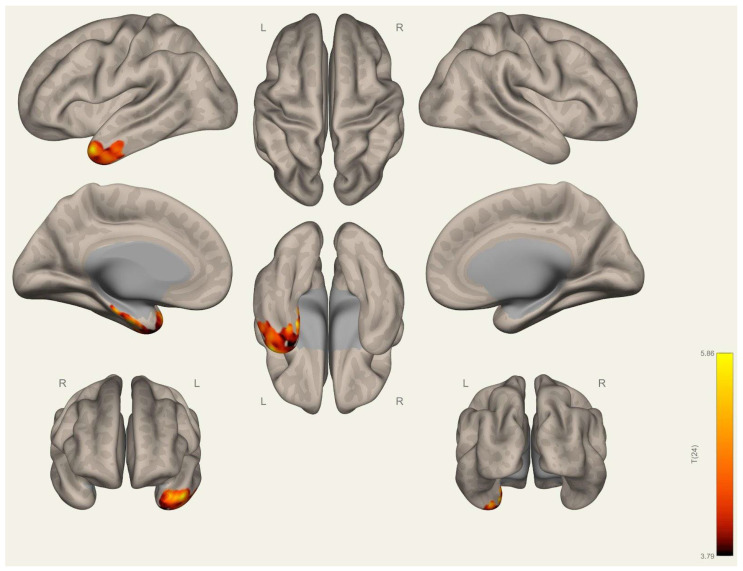
Group differences in functional connectivity at cluster-level corrected (*P*(_FWE_) < 0.05) for the “wrestlers > handball players” contrast (*Seed DMN MPFC*).

**Figure 4:**
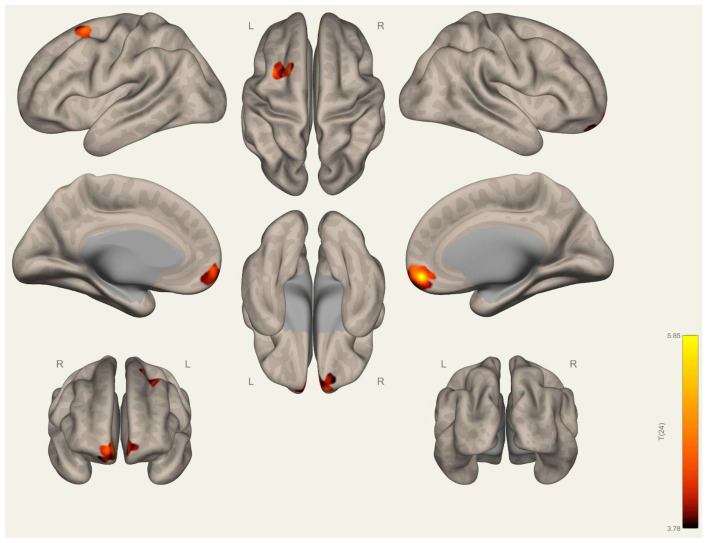
Group differences in functional connectivity at cluster-level corrected (*P*(_FWE_) < 0.05) for the “wrestlers > handball players” contrast (*Seed Visual Occipital*).

**Figure A4:**
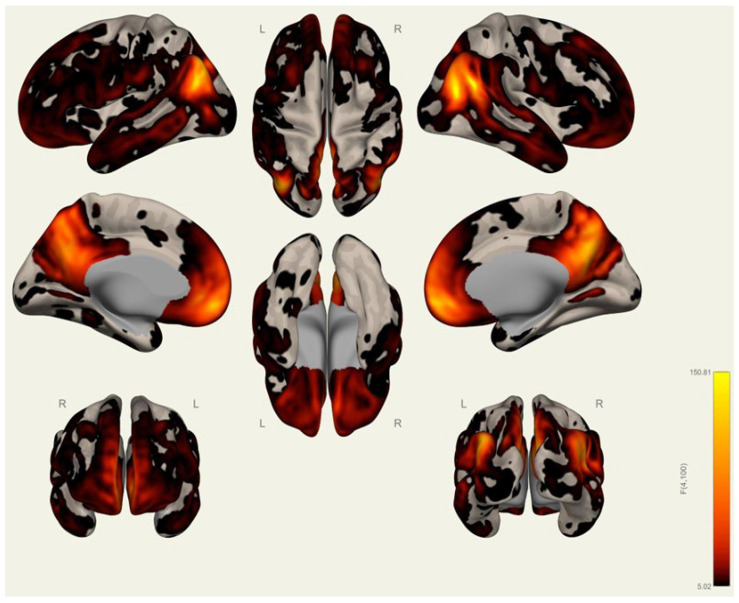
Resting-state default mode network map of handball players and wrestlers (*n* = 26, (*P*(_FWE_) < 0.05) extracted using the CONN toolbox in MATLAB. Lighter colours indicate the presence of the presence of stronger functional connectivity within the network.

**Figure A5:**
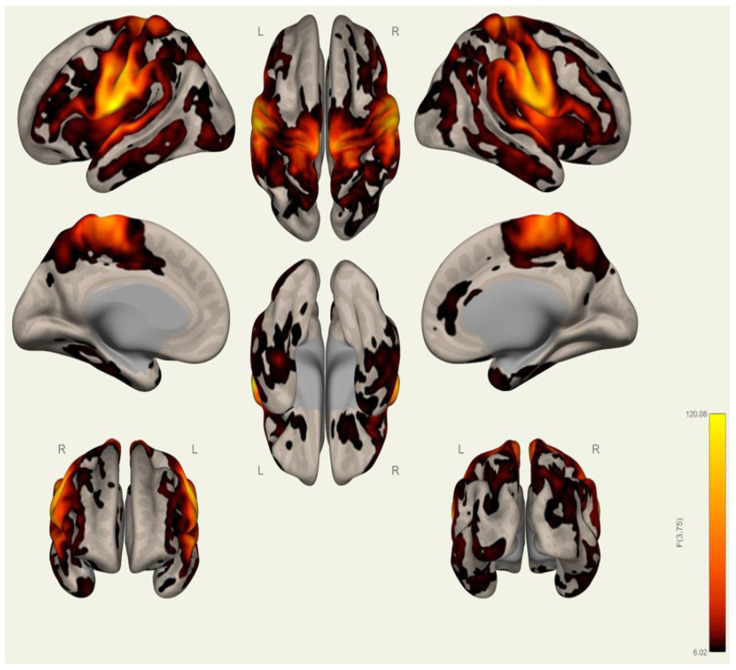
Resting-state somatosensory network map of handball players and wrestlers (*n* = 26, (*P*(_FWE_) < 0.05) extracted using the CONN toolbox in MATLAB. Lighter colours indicate the presence of stronger functional connectivity within the network.

**Figure A6:**
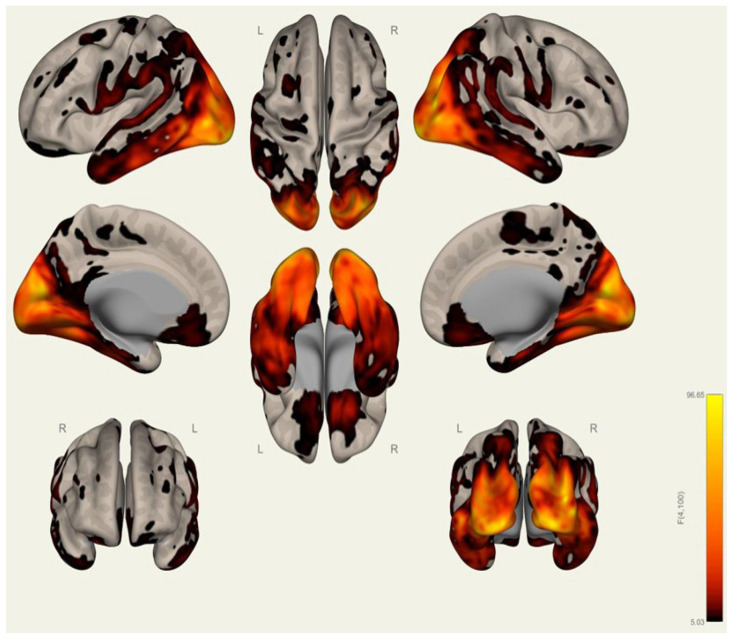
Resting-state visual network map of handball players and wrestlers (*n* = 26, (*P*(_FWE_) < 0.05) extracted using the CONN toolbox in MATLAB. Lighter colours indicate the presence of stronger functional connectivity within the network.

**Figure A7:**
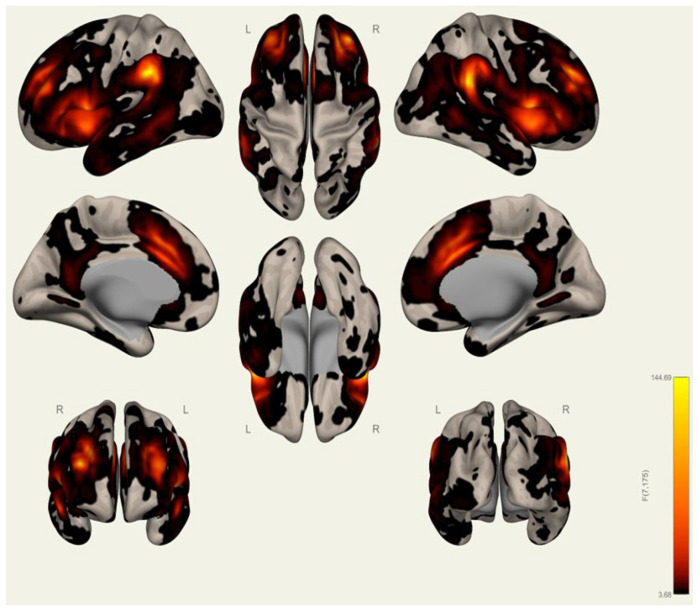
Resting-state salience network map of handball players and wrestlers (*n* = 26, (*P*(_FWE_) < 0.05) extracted using the CONN toolbox in MATLAB. Lighter colours indicate the presence of stronger functional connectivity within the network.

**Figure A8:**
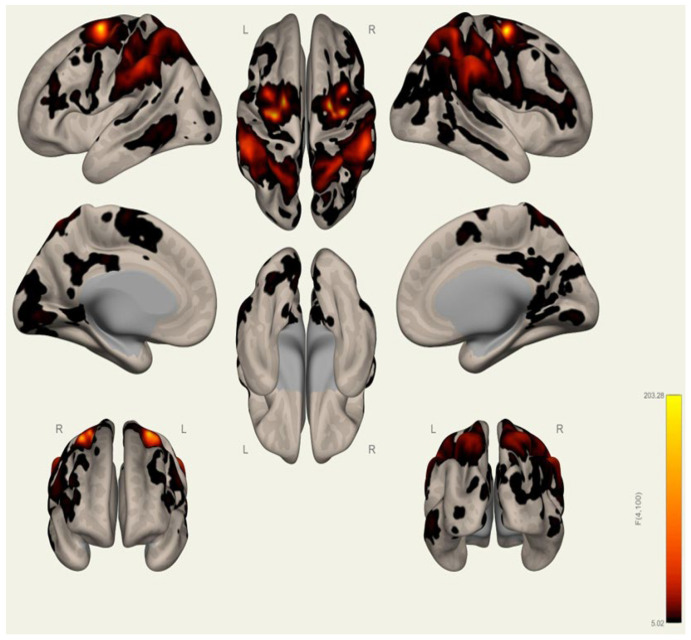
Resting-state dorsal attention network map of handball players and wrestlers (*n* = 26, (*P*(_FWE_) < 0.05) extracted using the CONN toolbox in MATLAB. Lighter colours indicate the presence of stronger functional connectivity within the network.

**Figure A9:**
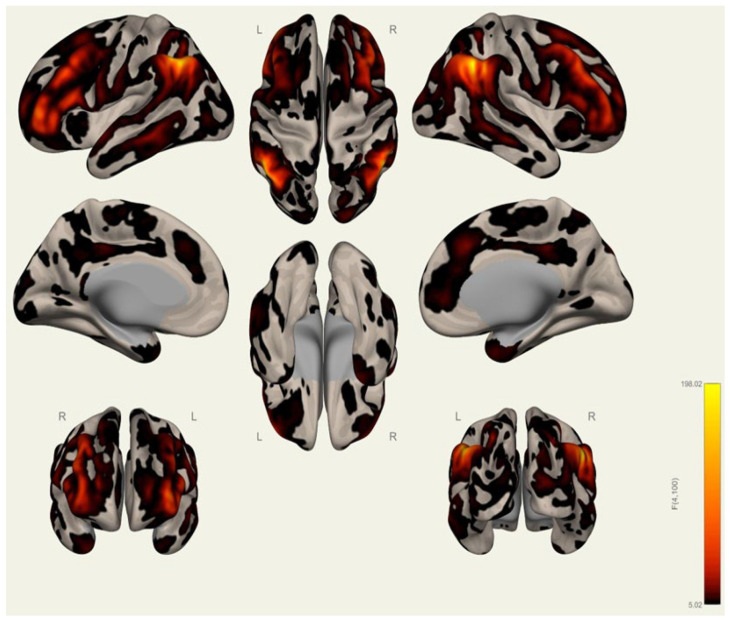
Resting-state frontoparietal network map of handball players and wrestlers (*n* = 26, (*P*(_FWE_) < 0.05) extracted using the CONN toolbox in MATLAB. Lighter colours indicate the presence of stronger functional connectivity within the network.

**Figure A10:**
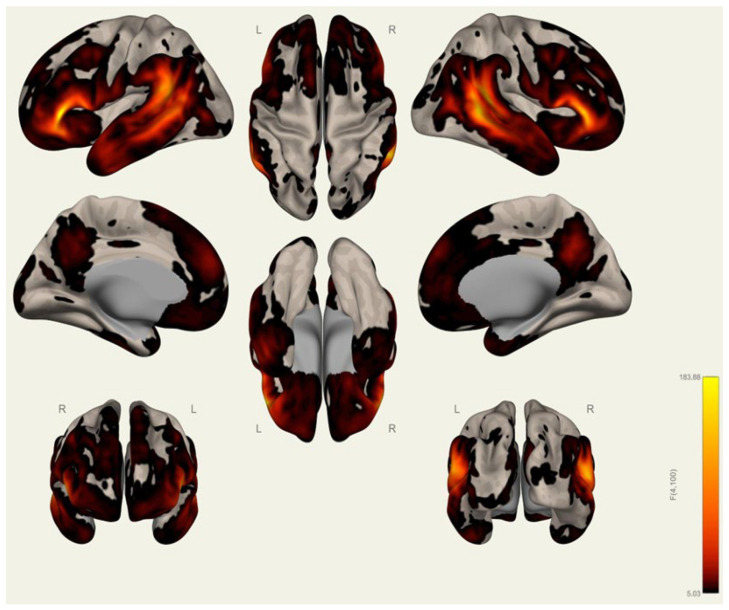
Resting-state language network map of handball players and wrestlers (*n* = 26, (*P*(_FWE_) < 0.05) extracted using the CONN toolbox in MATLAB. Lighter colours indicate the presence of stronger functional connectivity within the network.

**Figure A11:**
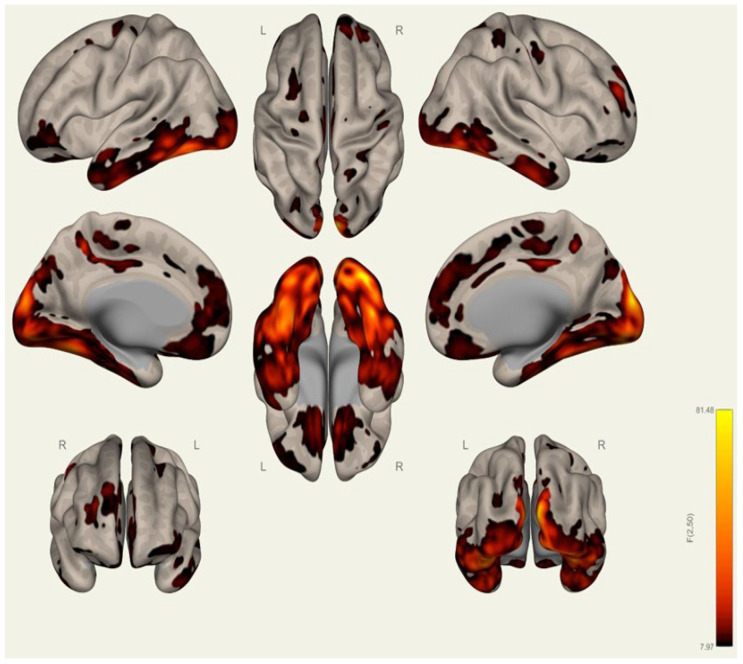
Resting-state cerebellar network map of handball players and wrestlers (*n* = 26, (*P*(_FWE_) < 0.05) extracted using the CONN toolbox in MATLAB. Lighter colours indicate the presence of stronger functional connectivity within the network.

**Figure A12:**
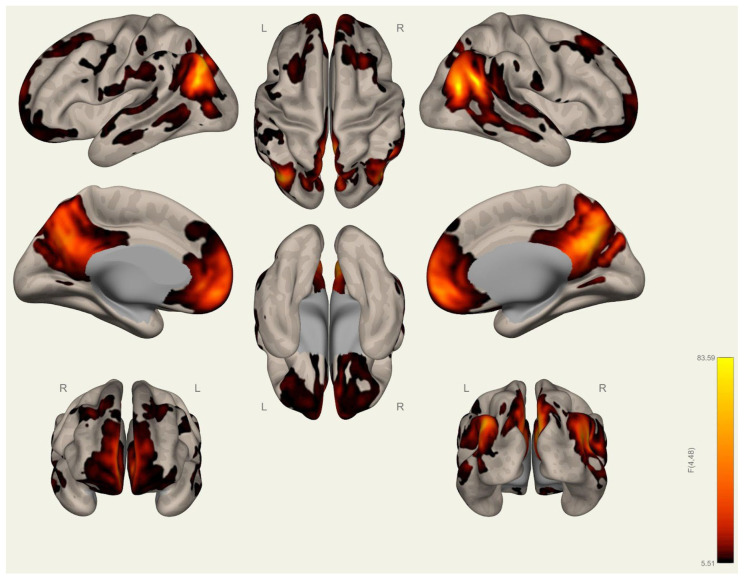
Resting-state default mode network map of handball players (*n* = 13, (*P*(_FWE_) < 0.05) extracted using the CONN toolbox in MATLAB. Lighter colours indicate the presence of stronger functional connectivity within the network.

**Figure A13:**
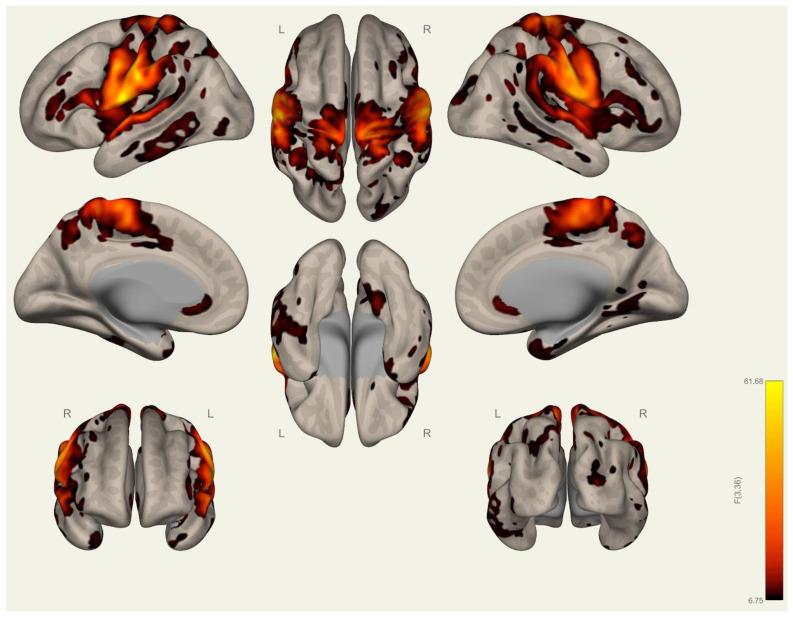
Resting somatosensory network map of handball players (*n* = 13, (*P*(_FWE_) < 0.05) extracted using the CONN toolbox in MATLAB. Lighter colours indicate the presence of stronger functional connectivity within the network.

**Figure A14:**
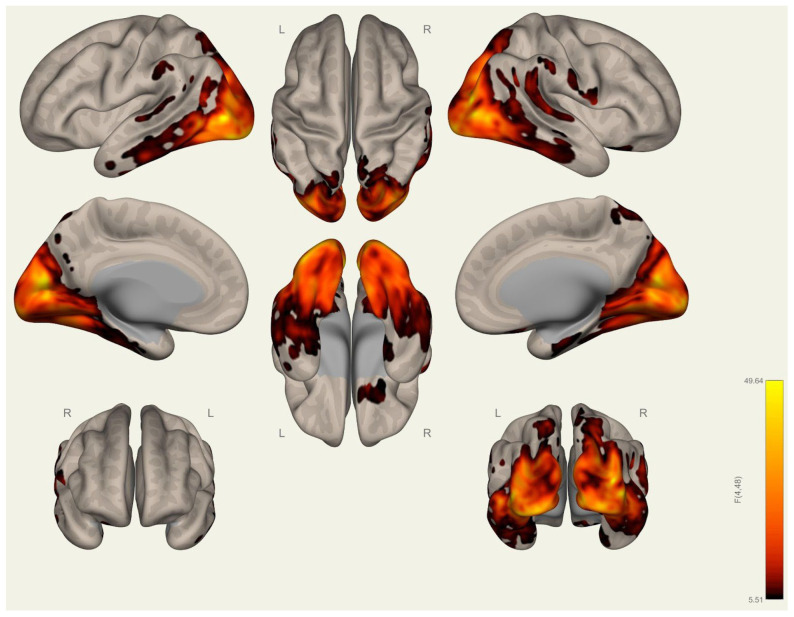
Resting-state visual network map of handball players (*n* = 13, (*P*(_FWE_) < 0.05) extracted using the CONN toolbox in MATLAB. Lighter colours indicate the presence of stronger functional connectivity within the network.

**Figure A15:**
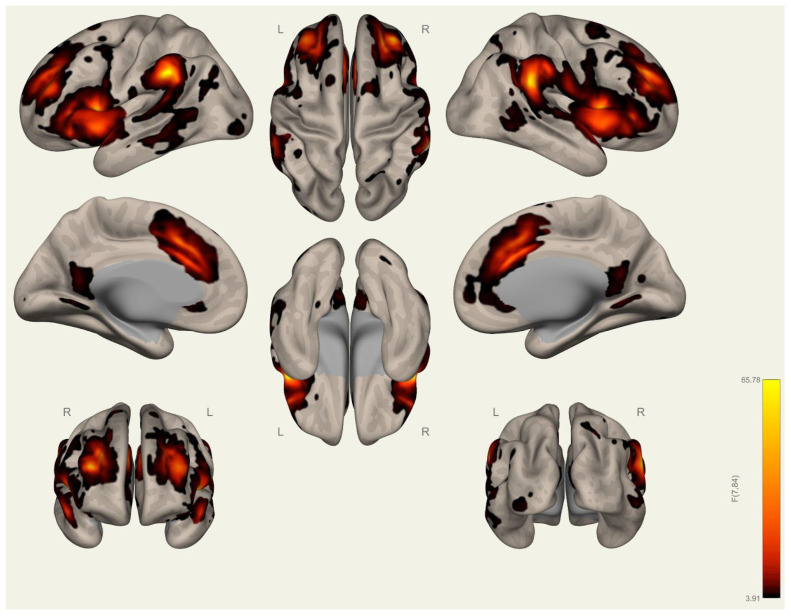
Resting-state salience network map of handball players (*n* = 13, (*P*(_FWE_) < 0.05) extracted using the CONN toolbox in MATLAB. Lighter colours indicate the presence of stronger functional connectivity within the network.

**Figure A16:**
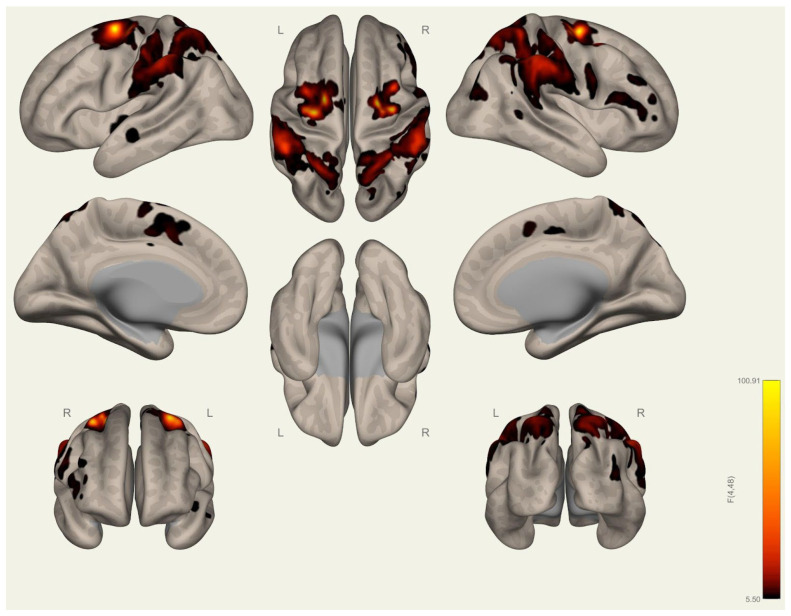
Resting-state dorsal attention network map of handball players (*n* = 13, (*P*(_FWE_) < 0.05) extracted using the CONN toolbox in MATLAB. Lighter colours indicate the presence of stronger functional connectivity within the network.

**Figure A17:**
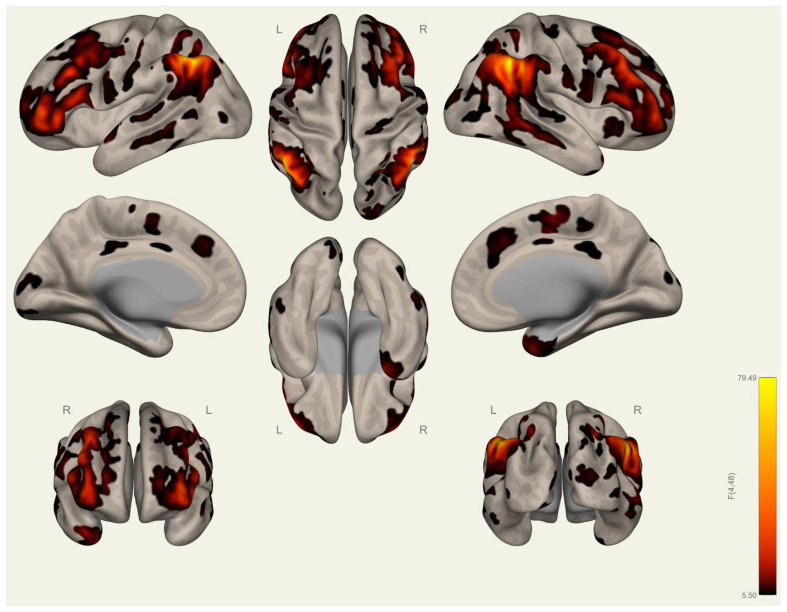
Resting-state frontoparietal network map of handball players (*n* = 13, (*P*(_FWE_) < 0.05) extracted using the CONN toolbox in MATLAB. Lighter colours indicate the presence of stronger functional connectivity within the network.

**Figure A18:**
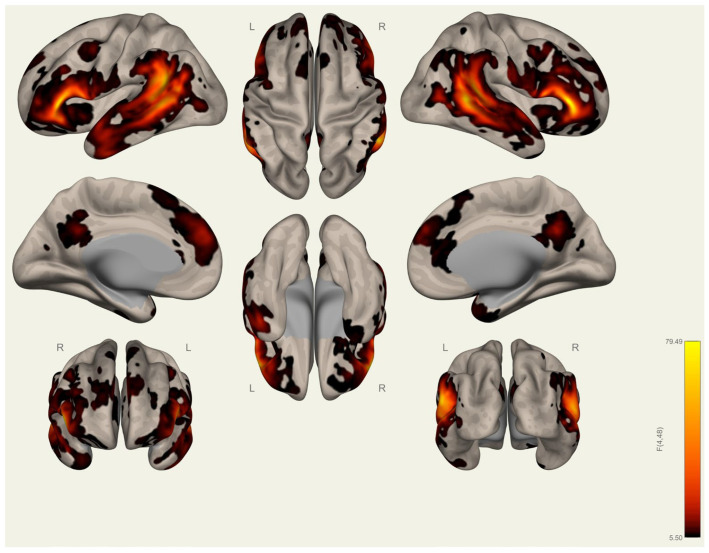
Resting-state language network nap of handball players (*n* = 13, (*P*(_FWE_) < 0.05) extracted using the CONN toolbox in MATLAB. Lighter colours indicate the presence of stronger functional connectivity within the network.

**Figure A19:**
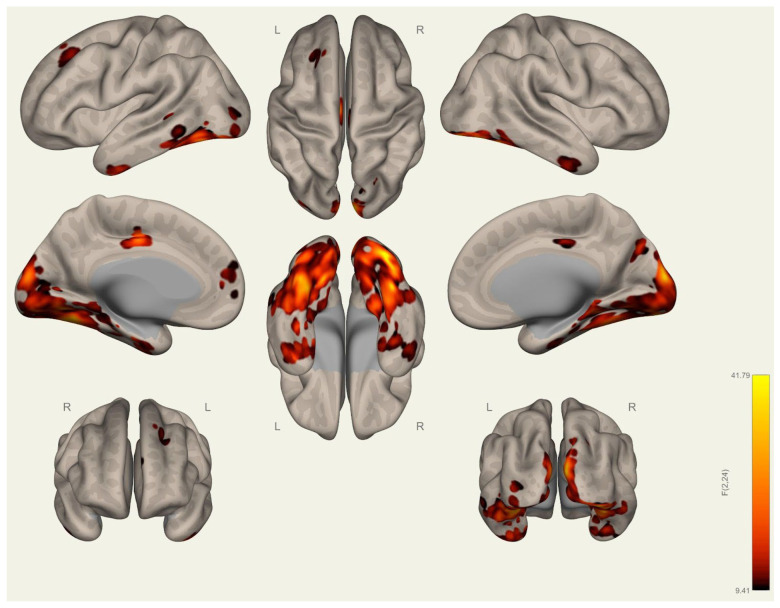
Resting-state cerebellar network map of handball players (*n* = 13, (*P*(_FWE_) < 0.05) extracted using the CONN toolbox in MATLAB. Lighter colours indicate the presence of stronger functional connectivity within the network.

**Figure A20:**
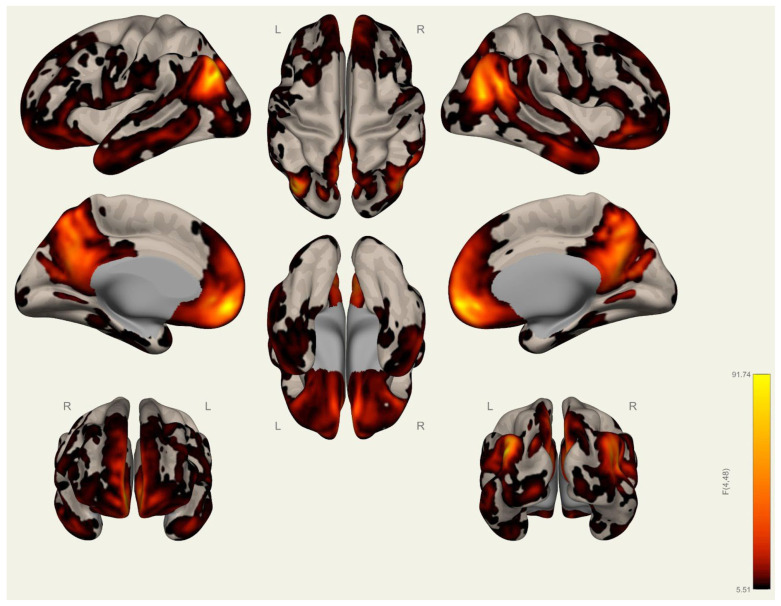
Resting-state default mode network map of wrestlers (*n* = 13, (*P*(_FWE_) < 0.05) extracted using the CONN toolbox in MATLAB. Lighter colours indicate the presence of stronger functional connectivity within the network.

**Figure A21:**
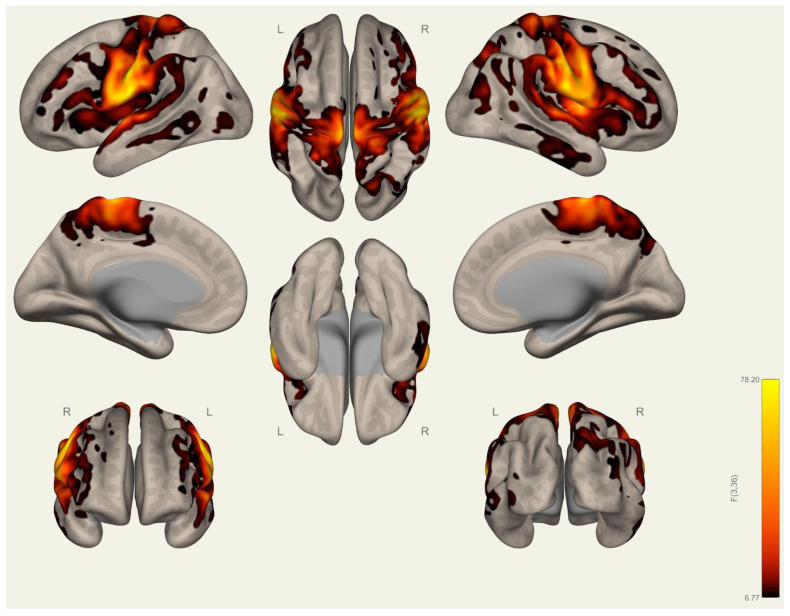
Resting somatosensory network map of wrestlers (*n* = 13, (*P*(_FWE_) < 0.05) extracted using the CONN toolbox in MATLAB. Lighter colours indicate the presence of stronger functional connectivity within the network.

**Figure A22:**
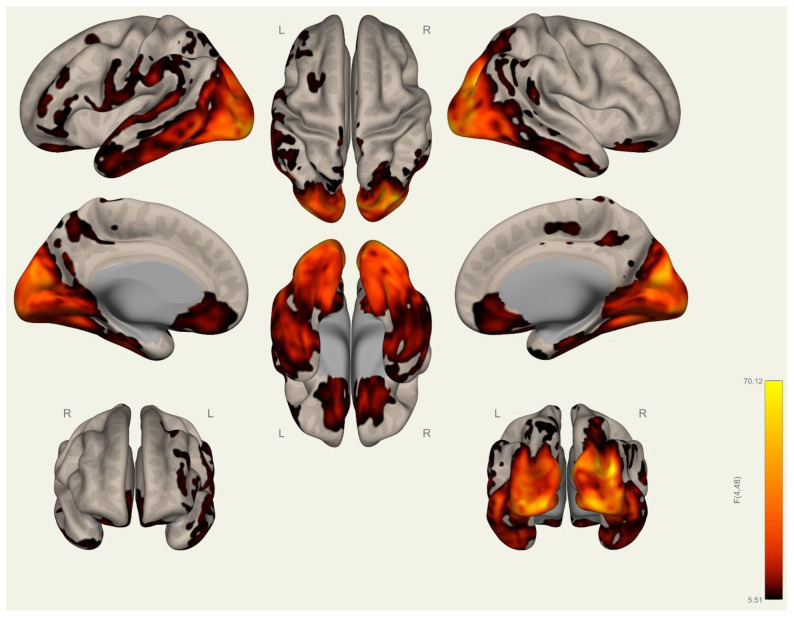
Resting-state visual network map of wrestlers (*n* = 13, (*P*(_FWE_) < 0.05) extracted using the CONN toolbox in MATLAB. Lighter colours indicate the presence of stronger functional connectivity within the network.

**Figure A23:**
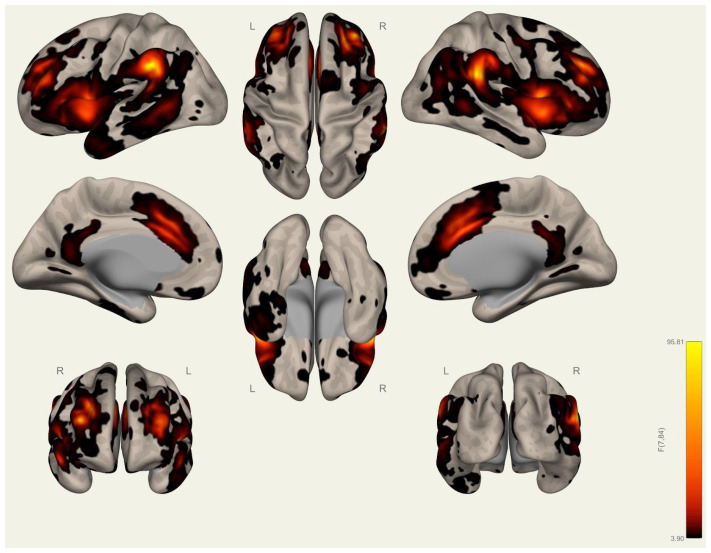
Resting-state salience network map of wrestlers (*n* = 13, (*P*(_FWE_) < 0.05) extracted using the CONN toolbox in MATLAB. Lighter colours indicate the presence of stronger functional connectivity within the network.

**Figure A24:**
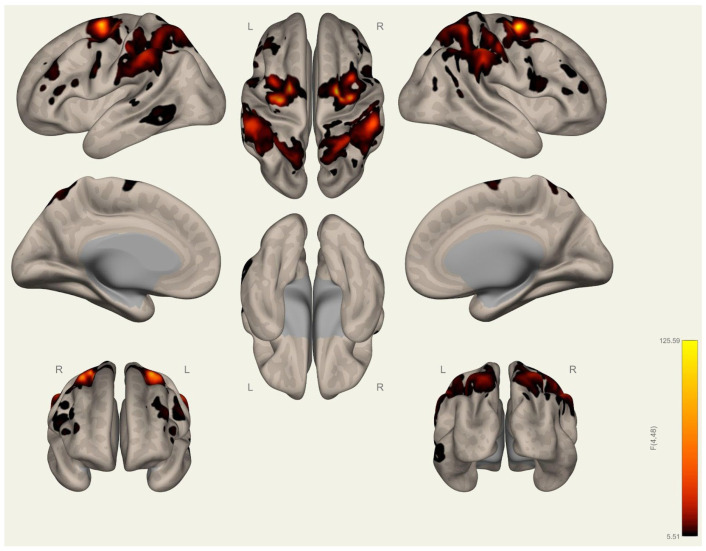
Resting-state dorsal attention network map of wrestlers (*n* = 13, (*P*(_FWE_) < 0.05) extracted using the CONN toolbox in MATLAB. Lighter colours indicate the presence of stronger functional connectivity within the network.

**Figure A25:**
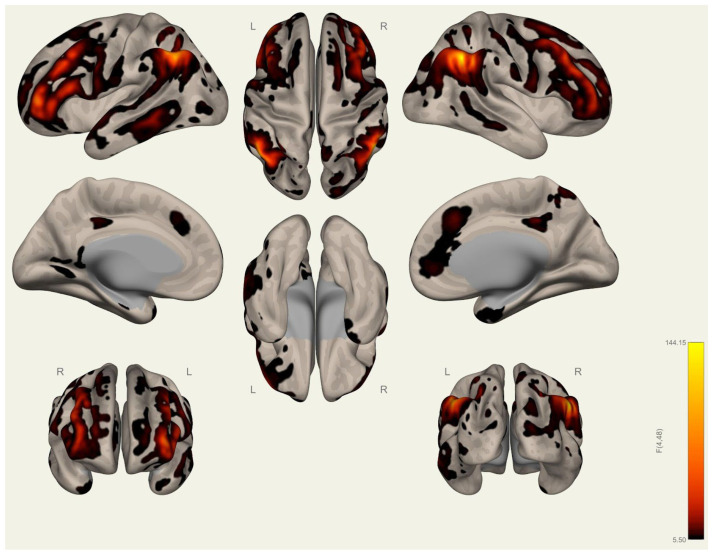
Resting-state frontoparietal network map of wrestlers (*n* = 13, (*P*(_FWE_) < 0.05) extracted using the CONN toolbox in MATLAB. Lighter colours indicate the presence of stronger functional connectivity within the network.

**Figure A26:**
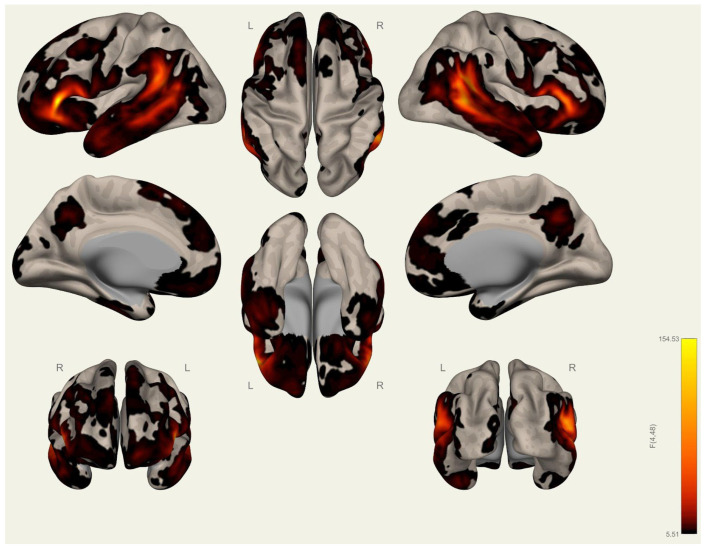
Resting-state language network map of wrestlers (*n* = 13, (*P*(_FWE_) < 0.05) extracted using the CONN toolbox in MATLAB. Lighter colours indicate the presence of stronger functional connectivity within the network.

**Figure A27:**
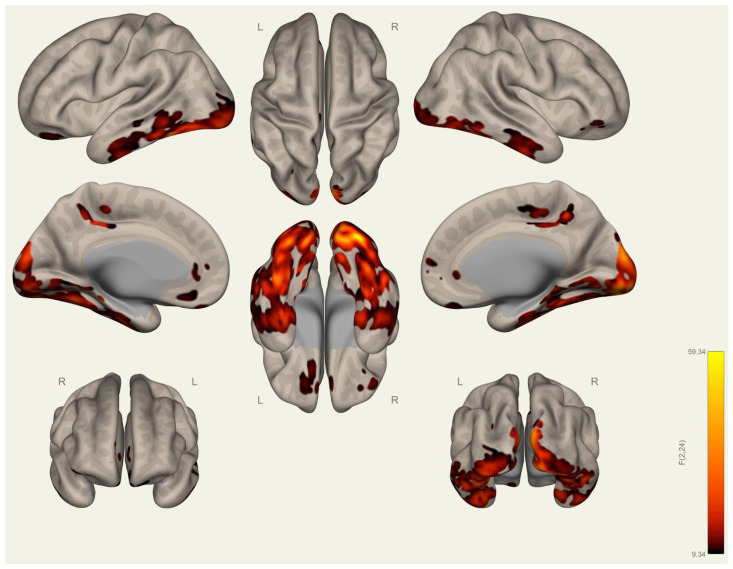
Resting-state cerebellar network map of wrestlers (*n* = 13, (*P*(_FWE_) < 0.05) extracted using the CONN toolbox in MATLAB. Lighter colours indicate the presence of stronger functional connectivity within the network.

Initially, the significant resting-state network maps are computed and visually evaluated. In line with the current state of the art [40,41], default mode, somatosensory, visual, salience, dorsal-attention, frontoparietal, language, and cerebellar networks are computed with a corrected threshold of PFWE < 0.05, and the results obtained are visualized for the participant group (*n* = 26) (Figure A4, Figure A5, Figure A6, Figure A7, Figure A8, Figure A9, Figure A10, Figure A11, Figure A12, Figure A13, Figure A14, Figure A15, Figure A16, Figure A17, Figure A18, Figure A19, Figure A20, Figure A21, Figure A22, Figure A23, Figure A24, Figure A25, Figure A26 and Figure A27 in Appendix A). Next, we investigated the differences in the resting-state networks between the wrestlers and handball players. Significant differences between the two groups are achieved when we apply the medial prefrontal cortex of the default mode network as a seed location. The left superior temporal gyrus (STG) and left parahippocampal gyrus (PHG) are found to significantly differ between the wrestlers and handball players after the cluster level correction (*P*(_FWE_) < 0.05) (Table 5). The significant differences are visualized in Figure 3. In addition to these findings, the left anterior orbital gyrus and right superior frontal gyrus–medial orbital regions are found to be significantly different when the visual occipital region is selected as the seed location (Figure 4).

## 4. Discussion

Numerous studies have demonstrated that prolonged engagement in professional physical exercise and motor skill acquisition induces structural and functional adaptations in the brain [22,42,43]. In the present study, we examined regional brain morphology differences between elite athletes in handball and wrestling. Our findings indicate that wrestlers exhibited significantly greater GMV in the right MTG, left MFG, and right PCG. In addition, wrestlers showed increased functional connectivity in the left STG and left PHG when the medial prefrontal cortex of the default mode network was a seed location. Furthermore, wrestlers showed increased functional connectivity in the left anterior orbital gyrus and right superior frontal gyrus–medial frontal region when the visual occipital region was selected as the seed. Additionally, wrestlers were shown to have greater cortical thickness values than handball players in many regions such as the left superior occipital sulcus, left transverse occipital sulcus, left temporal pole, right subcallosal gyrus, right medial orbital sulcus, right lateral orbitofrontal cortex (OFC), right PCG, middle PCG, and sulcus.

### 4.1. Regional Grey Matter Differences

The posterior cingulate cortex (PCC) is generally activated during tasks involving spatial coding, such as passive scene viewing, active navigation without response demands, spatial calculations, and self-localization within the environment. These regions have also been associated with internally directed cognition, including the retrieval of episodic and semantic memories [44,45]. Lesions in the right PCC are linked to topographic disorientation, a spatial cognition disorder, which is thought to stem from memory impairment rather than a deficit in perception [46]. Neurons in the PCC are related to monitoring eye movements in conjunction with visuospatial awareness, facilitating the localization of objects relative to the body [47]. Additionally, neurons in the PCC, which are activated by hand movements [48], are implicated in evaluating the state of the motor system rather than in direct motor control [47]. In our study, wrestlers exhibited greater GMV in the right PCG when compared to handball players.

Our findings indicate that wrestlers exhibit greater GMV in the left MFG compared to handball players. Previous research has highlighted the role of the left MFG in sports-related cognitive functions. For instance, a study by Xu et al. [49] demonstrated that badminton players showed heightened activation in the left MFG during tasks involving action prediction compared to novices, suggesting that this region facilitates athletes’ enhanced ability to anticipate and respond to dynamic sports actions. Additionally, a study in professional tennis players showed significantly increased left medial frontal gyrus activity in experts compared to novices, allowing them to focus on their fields, even in the presence of strong distractions [50]. Moreover, this region has been implicated in sustaining attention and managing cognitive control in tasks requiring focus and the suppression of distractions [51], as well as in processes related to memory retrieval, monitoring, and decision-making [52]. These findings collectively underscore the critical role of the left MFG in supporting the cognitive demands of specialized athletes.

In our study, wrestlers exhibited a greater GM volume in the right MTG when compared to its value in handball players. A meta-analysis by Wang et al. [53] reported increased right MTG volume in patients with social anxiety disorder compared to healthy controls. Additionally, Wang et al. [54] identified a positive correlation between social anxiety and GM volume in the right MTG. Pluhar et al. [55] highlighted that athletes in individual sports are more prone to anxiety and depression than those in team sports. Similarly, a study conducted on 15 elite males who participated in the United States Junior World Wrestling Camp in 1979 indicated that wrestlers had higher average attributional-state anxiety and tension psychologically [56]. The greater GM volume observed in the right MTG in our study may reflect the heightened anxiety commonly associated with individual sports such as wrestling.

Research indicates that the right MTG, in conjunction with the medial temporal lobe, contributes to the formation of new associations and concepts, particularly during creative tasks involving novelty and utility [57]. In wrestling, athletes frequently face novel and unpredictable situations that demand rapid thinking, decision-making, and the creative application of learned strategies to new contexts during competitions. The increased GMV observed in the right MTG of wrestlers may reflect its role in facilitating creative associations, enabling athletes to adapt to unexpected challenges and maintain performance. However, it remains unclear whether the elevated GMV in the right MTG is specifically linked to its creative association function or social anxiety. Further neuroimaging studies incorporating creativity-based tasks across various sports disciplines are needed to clarify this relationship.

### 4.2. Cortical Thickness Differences

A study demonstrated that elite long jumpers exhibited greater GMV in the left fusiform gyrus compared to aspiring long jumpers and elite javelin throwers. The authors attributed this finding to the high-precision visual tracking required during the approach phase of long jumpers, particularly in relation to the athlete’s body position and individual step patterns when approaching the takeoff board [58]. While the majority of research on the fusiform gyrus has focused on its role in face-selective activity, Peelen and Downing [35] observed that the right fusiform gyrus also contributes to the visual processing of the human body. Furthermore, Xu et al. [49] found that the left medial frontal cortex was functionally connected to the fusiform gyrus during the anticipation of skilled actions in professional badminton players, in contrast to novices. According to the theory proposed by Jung and Haier [59], changes within a network of critical brain regions, including the fusiform gyrus, may underlie individual differences in intelligence and reasoning during problem-solving tasks. In light of these studies, the increased right and left fusiform cortical thickness observed in wrestlers compared to handball players may reflect the specific cognitive and motor demands of wrestling, such as rapid visual processing, enhanced spatial awareness, and the need for quick decision-making during close combat scenarios [60,61].

The temporal pole has been implicated in several higher-order cognitive functions, including the visual processing of complex objects and facial recognition, autobiographical memory retrieval, naming and word-object association, and socio-emotional processing [36]. Our findings indicate that cortical thickness in the left temporal pole is significantly greater in wrestlers compared to handball players.

The middle PCC is modulated by short-latency somatosensory signals that facilitate body orientation in space, primarily through connections with the caudal cingulate motor area. Additionally, due to its nociceptive responses, it is suggested that the middle PCC mediates skeletal–motor orientation to noxious stimuli without engaging autonomic or emotional processing [62,63]. Our findings indicate that wrestlers exhibit greater cortical thickness in the middle PCC compared to handball players.

The subcallosal cingulate cortex plays a pivotal role in the regulation of mood and is notably hyperactive in individuals diagnosed with major depressive disorder [64]. This region is also thought to function as an interface between emotional and physiological states, mediating autonomic responses and contributing to the manifestation of mood disorders through physical symptoms, such as alterations in heart rate and endocrine function [65]. In our study, wrestlers exhibited greater cortical thickness in the subcallosal cingulate cortex compared to handball players. This finding may suggest that increased cortical thickness in this region is associated with athletes who are subjected to intense stress during short, high-pressure intervals, as commonly experienced in wrestling competition.

The right lateral OFC is implicated in the regulation of impulsive behaviours by assessing the potential consequences of actions, particularly those associated with risk, and evaluating the negative outcomes of prior decisions. It plays a central role in suppressing instinctive, reward-driven behaviours and assists in adjusting behaviour based on changing reward contingencies [66,67]. This region is essential in decision-making processes that involve weighing risks and rewards, particularly in learning from negative feedback, such as avoiding behaviours previously associated with punishment [68,69,70]. The observation of increased cortical thickness in the right OFC of wrestlers compared to handball players suggests that this region may be more frequently engaged in professional wrestlers, who must consistently avoid risky actions and adapt strategies in response to changing conditions during competition.

### 4.3. Functional Connectivity Differences

A study by Schlaffke et al. [23] demonstrated that endurance athletes exhibit greater GMV in the hippocampus, PHG, and medial temporal lobe compared to martial arts athletes. In the present study, a significant difference in functional connectivity was observed in the left PHG in wrestlers, a combat sport group, compared to handball players. The heightened parahippocampal functional connection found in wrestlers likely reflects the sport-specific cognitive demands of wrestling, including spatial navigation, memory encoding, contextual associations, and sensorimotor integration in dynamic and physically demanding situations [71,72,73,74,75].

The STS is recognized as one of the core structures involved in the theory of mind and is implicated in various cognitive processes, including audiovisual integration, biological motion perception, speech processing, and face perception [37,76]. Moreover, research has demonstrated that this region not only processes biological motion but also encodes how another individual’s movements relate to their intentions [77]. A study by Wang et al. [78] indicated that poorer sleep quality is associated with reduced cortical thickness in the left STS, suggesting a potential neural substrate for the link between anxiety, depressive symptoms, and sleep disturbances. fMRI research on the mirror neuron system also suggests that the STS actively represents visuomotor relationships between observed actions and the observer’s own movements, beyond passively registering biological motion [79]. Additionally, studies have reported increased cortical thickness in the right PHG, right OFC, and left STS in experienced taxi drivers compared to controls [8,23]. Our findings revealed enhanced functional connectivity in the left STS in wrestlers compared to handball players. The enhanced functional connectivity in the STS of wrestlers may support their superior capacity for social cognition, biological motion processing, and multisensory integration, all of which are crucial for optimizing performance in the high-pressure, real-time environment of wrestling. These brain adaptations may result from the intense physical and cognitive demands specific to sports in regions involved in expertise in motor and cognitive tasks.

A study by Jin et al. [2] demonstrated that skeleton athletes exhibited stronger functional connectivity between the superior frontal gyrus and the insula—brain regions associated with cognitive and motor control—and areas involved in visual processing, reward learning, spatial cognition, and emotional processing during resting-state brain function, compared to controls. Zhang et al. [25] investigated brain activity in volleyball athletes using resting-state fMRI and reported decreased activity in the right superior frontal gyrus compared to the control group. Zhang et al. proposed that these findings reflect functional adaptations resulting from extensive sports training, potentially indicative of increased neural efficiency in motor and cognitive processes. In contrast, our findings reveal higher right superior frontal gyrus activation in wrestlers compared to handball players. This observation suggests two possible interpretations: first, the right superior frontal gyrus may be more functionally developed in handball players, leading to greater neural efficiency compared to wrestlers. Alternatively, this difference could represent an adaptation to the specific cognitive and motor demands of team sports, such as coordinated gameplay and rapid decision-making. However, given the absence of a control group in our study, these interpretations remain speculative and warrant further investigation in future research.

### 4.4. Integration of PPS Dynamics

PPS describes the region of space immediately surrounding the body, where objects can be grasped and manipulated, whereas extrapersonal space refers to the region beyond the body’s reach, where exploratory eye movements predominate [42]. These brain maps are informed primarily by sensory inputs from vision and touch. They are underpinned by various types of neurons that respond to visual and/or tactile stimuli generated by objects in close proximity to the body [80]. Although PPS was not the primary focus of this study, the observed differences in brain regions may still have implications for spatial processing in athletes.

In the team sport of handball, the ball is an object that moves away from the hand and returns to the hand, while in the individual branch of wrestling, the opponent’s body acts as an object, remaining in constant contact with the hand and being in close proximity to the athlete’s body. In handball, the athlete assesses actions occurring at a greater distance from their body, whereas in wrestling, they closely monitor the contest occurring in immediate proximity to their body. A commonality between these sports is that both the visual system and the pro-prioceptive/tactile systems are actively engaged during the athletes’ performance. These differing spatial demands suggest varying reliance on visual, proprioceptive, and tactile systems. In this context, we observed significant differences in regions such as the PCC, middle PCC, STS, MTG, OFC, and PHG—areas involved in spatial representation and body awareness. However, we did not find differences in primary PPS-related regions such as the ventral premotor cortex, intraparietal sulcus, parietal cortex, insula, putamen, or postcentral gyrus [39,81,82,83].

Although the PCC is not directly responsible for the encoding process of PPS, its role in spatial awareness and memory likely provides a higher-order context for interactions within this space. The PCC plays a critical role in the integration of spatial information to form a coherent understanding of one’s position within the environment, a function essential for large-scale navigation rather than immediate spatial processing in PPS. Similarly, the OFC is not the primary locus for PPS processing; it may play an indirect role by supporting adaptive behaviour responses based on the value and potential consequences of interacting with objects or threats within PPS [38,84]. Furthermore, the OFC interacts with other brain regions, such as the parietal and premotor cortices, which are more directly involved in spatial mapping and motor responses related to PPS. Within this network, the OFC primarily contributes to the evaluative and motivational aspects of interactions [85].

While the STS is not directly involved in the processing of PPS, its role in social perception, multisensory integration, and biological motion perception may indirectly influence responses to stimuli within the PPS. The MTG is involved in higher-level visual processing, particularly regarding motion, object recognition, and the integration of sensory information [86]. These functions may contribute to spatial awareness and the ability to interpret dynamic interactions within the PPS.

Moreover, recent studies suggest that the PHG, known for its role in spatial memory and scene processing, may also be involved in encoding the spatiotemporal dimensions of PPS, particularly in integrating multisensory information from the surrounding environment [38,81]. However, this connection remains relatively novel and is less established compared to the involvement of regions such as the premotor and parietal cortices. Additionally, although the middle PCC is not central to PPS processing, it may indirectly contribute to PPS-related functions through its involvement in action planning and sensory–motor integration in response to environmental stimuli.

While no direct neural correlates of PPS were identified, we observed that wrestlers exhibited greater GMV, cortical thickness, and functional connectivity in regions indirectly related to PPS, such as the STG, PHG, and PCG. This heightened involvement of peripersonal-related regions in wrestlers may reflect the sport’s reliance on precise body positioning, an awareness of opponents in proximity, and rapid motor adjustments during physical combat. In contrast, handball players, whose sport emphasizes coordinated team play and spatial strategies over direct physical contact, showed comparatively less pronounced adaptations in these regions.

Wrestling is a physically intense sport involving frequent head and neck engagement, including maneuvers such as hand-to-neck pulls, headlocks, and takedowns, which expose athletes to repetitive impact over years of training. Given this, one might question whether the increased GMV and cortical thickness observed in wrestlers could be related to cumulative concussive or sub-concussive impacts rather than sport-specific neuroplasticity. However, existing research does not consistently associate repetitive head impacts or sports-related concussions with increased GMV or cortical thickness. Studies examining the structural effects of repetitive head impacts have primarily reported reductions in GM, particularly in the hippocampus [87,88], or found no significant volumetric changes at all [89]. Similarly, research on cortical thickness in athletes with a history of sports-related concussions often indicates cortical thinning rather than thickening [90]. In a study conducted by Oliveira et al. [91], neither heading nor concussion in soccer players was associated with reductions in brain volume or cortical thickness. The authors observed a positive association between increased heading frequency and greater GMV in the left inferior parietal region. However, they concluded that this finding likely reflects neuroplastic adaptations related to the acquisition of heading skills rather than the direct effects of repetitive head impacts. Longitudinal studies have also demonstrated that exposure to head impacts generally leads to decreased cerebral blood flow over time rather than structural increases in GM [92]. These findings suggest that the greater GMV and cortical thickness observed in wrestlers in the present study are unlikely to be attributable to head impacts. Instead, they may be better explained by sport-specific neuroplastic adaptations, reinforcing the role of intense, sustained motor training in shaping brain structure.

In this study, we utilized a 1.5 Tesla MRI scanner to acquire anatomical and functional images. However, the use of 1.5T MRI presents certain limitations compared to 3T or higher-field scanners. One major drawback is the reduced signal-to-noise ratio (SNR), which may affect image quality. Additionally, higher magnetic field strengths allow for submillimeter resolution, whereas 1.5T MRI offers a comparatively lower spatial resolution. Despite this, the resolution obtained in our study is considered sufficient for comparing the two groups. Another limitation pertains to the BOLD signal. Lower field strengths are more susceptible to physiological noise, such as cardiac pulsations and respiratory fluctuations. However, in our study, the athletes’ lower resting pulse rates may have mitigated this effect to some extent.

As both participant groups consisted of elite athletes, heart rate variability and respiratory variability were not considered in the comparison between the two groups. On the other hand, excluding these parameters in the analysis can be considered a limitation of this study.

These findings highlight the distinct neuroplastic responses elicited by the specific motor and cognitive demands of each sport. While we recognize that genetic predispositions and lifestyle factors may contribute to structural brain differences, our study primarily focuses on training-induced neuroplastic adaptations. Future research incorporating genetic profiling and comprehensive lifestyle assessments will be essential in disentangling these influences. Moreover, the lack of a control group and the cross-sectional design limit our ability to establish causality or determine whether these adaptations are sport-specific or general effects of athletic training. Longitudinal studies that track athletes over time and employ targeted functional assessments are warranted to determine how these structural adaptations translate into cognitive and athletic performance. A deeper understanding of these mechanisms could contribute to optimizing training regimens and refining athlete development strategies in various sports disciplines.

## 5. Conclusions

This study demonstrated the influence of sport-specific training on brain structure and function by uncovering distinct neural adaptations in wrestlers compared to handball players. The findings underscore the plasticity of the human brain in response to the differing physical and cognitive demands of these sports, highlighting potential implications for motor control, spatial cognition, and cognitive performance. Beyond theoretical insights, these findings may inform practical applications in coaching, athlete selection, and injury prevention. Identifying functionally and morphologically altered brain regions in professional athletes may provide new avenues for modulating neuroplasticity to enhance sport-specific skills. Future research incorporating control groups, genetic and lifestyle assessments, and longitudinal designs will be crucial to further elucidate the interplay between training, neuroplasticity, and performance.

## Figures and Tables

**Table 1:** ROI clusters for CONN’s networks.

Network	ROI	Coordinate
Cerebellar	Anterior	(0,−63,−30)
Cerebellar	Posterior	(0,−79,−32)
Frontoparietal	LPFC (L)	(−43,33,28)
Frontoparietal	PPC (L)	(−46,−58,49)
Frontoparietal	LPFC (R)	(41,38,30)
Frontoparietal	PPC (R)	(52,−52,45)
Default mode	MPFC	(1,55,−3)
Default mode	LP (L)	(−39,−77,33)
Default mode	LP (R)	(47,−67,29)
Default mode	PCC	(1,−61,38)
Sensorimotor	Lateral (L)	(−55,−12,29)
Sensorimotor	Lateral (R)	(56,−10,29)
Sensorimotor	Superior	(0,−31,67)
Dorsal attention	FEF (L)	(−27,−9,64)
Dorsal attention	FEF (R)	(30,−6,64)
Dorsal attention	IPS (L)	(−39,−43,52)
Dorsal attention	IPS (R)	(39,−42,54)
Language	IFG (L)	(−51,26,2)
Language	IFG (R)	(54,28,1)
Language	pSTG (L)	(−57,−47,15)
Language	pSTG (R)	(59,−42,13)
Salience	ACC	(0,22,35)
Salience	AInsula (L)	(−44,13,1)
Salience	AInsula (R)	(47,14,0)
Salience	RPFC (L)	(−32,45,27)
Salience	RPFC (R)	(32,46,27)
Salience	SMG (L)	(−60,−39,31)
Salience	SMG (R)	(62,−35,32)
Visual	Medial	(2,−79,12)
Visual	Occipital	(0,−93,−4)
Visual	Lateral (L)	(−37,−79,10)
Visual	Lateral (R)	(38,−72,13)

ROIs used in the functional connectivity analysis. The table presents the ROI clusters for CONN’s networks, including their respective functional network classifications and MNI coordinates. These ROIs were selected based on the 164 HPC-ICA parcellation and served as seed locations for connectivity analyses.

**Table 2:** Mann–Whitney U test results for the group comparison (H0, μ wrestling = μ handball).

Parameter	Group	Mean	Median	SD	U	*p*	SE
CSF absolute volume cm^3^	Wrestling	237.77	259.00	76.91	69	0.442	19.5
	Handball	263.62	262.00	48.68			
CSF relative volume (%)	Wrestling	15.98	16.20	1.99	69	0.442	19.5
	Handball	16.77	16.90	2.71			
GM absolute volume	Wrestling	761.85	751.00	63.97	62	0.259	19.5
	Handball	729.23	722.00	52.64			
GM relative volume (%)	Wrestling	48.28	47.40	1.75	37	0.016	19.5
	Handball	46.55	46.30	1.73			
WM absolute volume	Wrestling	564.85	559.00	56.98	67	0.383	19.5
	Handball	573.92	570.00	45.48			
WM relative volume (%)	Wrestling	35.74	35.50	1.19	67.5	0.397	19.5
	Handball	36.67	35.90	2.41			
TIV	Wrestling	1580.08	1541.00	148.10	82	0.918	19.5
	Handball	1566.77	1564.00	100.67			
Thickness	Wrestling	2.45	2.47	0.09	64	0.304	19.5
	Handball	2.42	2.44	0.07			

Mann–Whitney U test results for the group comparison of anatomical brain structures between wrestlers and handball players. The table presents the mean, median, standard deviation (SD), and standard error (SE) along with U and *p*-values. Significant differences (*p* < 0.05) indicate variations in cortical thickness and volumetric parameters between the two groups. CSF: cerebrospinal fluid; GM: grey matter; WM: white matter; TIV: total intracranial volume.

**Table 3:** Group differences in anatomical connectivity at cluster-level corrected *P*(_FWE_) = 0.05 for the “wrestlers > handball players” contrast.

Brain Region	(x, y, z)	Peak T	Peak *p*	Peak *p*-FWE
Wrestling > handball				
Middle temporal gyrus (R)	60 −6 −27	4.41	0.0001	0.22
	61.5 −15 −21	3.68	0.0006	0.67
Middle frontal gyrus (L)	−27 0 57	4.20	0.0002	0.32
Posterior cingulate gyrus (R)	10.5 −30 43.5	3.60	0.0007	0.72

The table presents significant regions identified in the ‘wrestlers > handball’ contrast, with their peak MNI coordinates (x, y, z), corresponding T-values, and uncorrected *p*-values (*p* < 0.001) with an extent threshold of 10 voxels. Significant clusters were observed in the middle temporal gyrus, middle frontal gyrus, and posterior cingulate gyrus, indicating structural differences in connectivity.

**Table 4:** Mann–Whitney U test results for cortical thickness differences in brain regions of handball players and wrestlers (H0, μ Wrestling = μ Handball).

Brain Region	Group	Mean	Median	SD	U	*p*	*SE*
Left superior occipital sulcus and transverse occipital sulcus	Wrestling	2.07	2.06	0.10	43.0	0.034	19.5
	Handball	1.98	1.94	0.10			
Left superior temporal sulcus	Wrestling	2.41	2.41	0.09	30.5	0.006	19.5
	Handball	2.33	2.32	0.01			
Left fusiform	Wrestling	2.69	2.68	0.07	41.0	0.027	19.5
	Handball	2.61	2.61	0.08			
Right fusiform	Wrestling	2.74	2.73	0.10	37.0	0.014	19.5
	Handball	2.62	2.63	0.10			
Left temporal pole	Wrestling	3.63	3.61	0.15	38.0	0.016	19.5
	Handball	3.33	3.36	0.36			
Right middle–posterior part of the cingulate gyrus and sulcus	Wrestling	2.61	2.62	0.11	35.0	0.012	19.5
	Handball	2.50	2.47	0.08			
Right subcallosal area, subcallosal gyrus	Wrestling	2.77	2.84	0.20	34.0	0.010	19.5
	Handball	2.56	2.56	0.28			
Right medial orbital sulcus (olfactory sulcus)	Wrestling	2.33	2.32	0.12	43.0	0.035	19.5
	Handball	2.20	2.14	0.19			
Right lat. orbitofrontal	Wrestling	2.73	2.73	0.07	45.5	0.048	19.5
	Handball	2.64	2.68	0.11			
Right posterior cingulate	Wrestling	2.55	2.54	0.10	35.0	0.012	19.5
	Handball	2.41	2.36	0.12			

Mann–Whitney U test results for cortical thickness differences between wrestlers and handball players. The table presents the mean, median, standard deviation (SD), and standard error (SE) along with U and *p*-values for each brain region. Significant differences (*p* < 0.05) indicate greater cortical thickness in several regions for wrestlers, particularly in the occipital, temporal, frontal, and limbic areas.

**Table 5:** Group differences in functional connectivity at cluster-level corrected *P*(_FWE_) < 0.05 for the “wrestlers > handball players” contrast.

Brain Region	Cluster (x, y, z)	Cluster *p*-FWE	Peak *p*-FWE	Peak T
Wrestling > handball				
Seed DMN MPFC	*1 55 −3*			
Left superior temporal gyrus	−38 10 −32	0.001	0.022	6.82
Left parahippocampal gyrus	−24 −14 −34	0.020	0.049	6.40
Seed Visual Occipital	*0 −93 −4*			
Left anterior orbital gyrus	−26 40 −14	0.005	0.029	6.71
Right superior frontal gyrus–medial orbital	10 52 −14	0.002	0.035	6.62

## Data Availability

Dataset available on request from the authors.

## Abbreviations

The following abbreviations are used in this manuscript:

BOLD	Blood Oxygenation Level-Dependent
CSF	cerebrospinal fluid
DARTEL	diffeomorphic anatomical registration through exponentiated lie algebra
EPI	echo-planar imaging
FOV	field of view
fMRI	functional magnetic resonance imaging
GM	gray matter
GMV	gray matter volume
MRI	magnetic resonance imaging
MFG	middle frontal gyrus
MTG	middle temporal gyrus
OFC	orbitofrontal cortex
PHG	parahippocampal gyrus
PPS	peripersonal space
PCC	posterior cingulate cortex
PCG	posterior cingulate gyrus
TE	echo time
TR	repetition time
ROI	region of interest
STG	superior temporal gyrus
STS	superior temporal sulcus
VBM	voxel-based morphometry
WM	white matter

## Appendix A

Functional connectivity strength was represented by Fisher-transformed bivariate correlation coefficients from a weighted general linear model, defined separately for each pair of seed and target areas, modelling the association between their BOLD signal time series. For each individual voxel, a separate GLM was estimated, with first-level connectivity measured at this voxel serving as the dependent variables, and groups serving as independent variables. Voxel-level hypotheses were evaluated using multivariate parametric statistics with random effects across subjects. Inferences were performed at the level of individual clusters (groups of contiguous voxels). Cluster-level inferences were based on parametric statistics from Gaussian Random Field theory.

**Table A1:** Statistical values of ROI-to-ROI connectivity analysis for all subjects.

Analysis Unit			T (25)-Value	p-unc	p-FDR	*p*-FWE
Cluster 1/34						
	Score	1378.23		0.000000	0.000000	0.000000
	Mass	3402.43		0.000000	0.000000	0.000000
	Size	48		0.000000	0.000000	0.000000
DefaultMode LP (R) (47,−67,29)-DefaultMode LP (L) (−39,−77,33)			16.11	0.000000	0.000000	
Cerebellar Posterior (0,−79,−32)-Cerebellar Anterior (0,−63,−30)			14.69	0.000000	0.000000	
Visual Lateral (R) (38,−72,13)-Visual Medial (2,−79,12)			12.61	0.000000	0.000000	
Visual Lateral (R) (38,−72,13)-Visual Occipital (0,−93,−4)			11.85	0.000000	0.000000	
Visual Medial (2,−79,12)-Visual Occipital (0,−93,−4)			11.78	0.000000	0.000000	
Visual Lateral (R) (38,−72,13)-Visual Lateral (L) (−37,−79,10)			11.00	0.000000	0.000000	
Visual Lateral (L) (−37,−79,10)-Visual Occipital (0,−93,−4)			9.21	0.000000	0.000000	
Visual Lateral (L) (−37,−79,10)-Visual Medial (2,−79,12)			9.16	0.000000	0.000000	
Visual Occipital (0,−93,−4)-Cerebellar Posterior (0,−79,−32)			8.74	0.000000	0.000000	
DefaultMode.MPFC (1,55,−3)-DefaultMode LP (L) (−39,−77,33)			7.26	0.000000	0.000002	
DefaultMode.LP (R) (47,−67,29)-DefaultMode PCC (1,−61,38)			6.94	0.000000	0.000003	
DefaultMode LP (L) (−39,−77,33)-Visual Lateral (L) (−37,−79,10)			6.83	0.000000	0.000004	
DefaultMode LP (R) (47,−67,29)-DefaultMode MPFC (1,55,−3)			6.51	0.000001	0.000007	
DefaultMode LP (R) (47,−67,29)-Visual Lateral (R) (38,−72,13)			6.26	0.000002	0.000013	
DefaultMode MPFC (1,55,−3)-DefaultMode PCC (1,−61,38)			5.62	0.000008	0.000055	
Visual Occipital (0,−93,−4)-Cerebellar Anterior (0,−63,−30)			5.37	0.000015	0.000092	
DefaultMode PCC (1,−61,38)-DefaultMode LP (L) (−39,−77,33)			5.36	0.000015	0.000092	
Visual Medial (2,−79,12)-Cerebellar Posterior (0,−79,−32)			5.09	0.000030	0.000176	
Visual Medial (2,−79,12)-Cerebellar Anterior (0,−63,−30)			4.51	0.000131	0.000619	
Visual Lateral (R) (38,−72,13)-Cerebellar Anterior (0,−63,−30)			3.97	0.000537	0.002134	
DefaultMode LP (L) (−39,−77,33)-Visual Lateral (R) (38,−72,13)			3.95	0.000569	0.002207	
Visual Lateral (L) (−37,−79,10)-Cerebellar Anterior (0,−63,−30)			3.36	0.002532	0.007817	
Visual Lateral (R) (38,−72,13)-Cerebellar Posterior (0,−79,−32)			3.19	0.003778	0.010782	
Visual Lateral (L) (−37,−79,10)-Cerebellar Posterior (0,−79,−32)			2.17	0.039513	0.076421	
Cluster 2/34						
	Score	1146.55		0.000000	0.000000	0.000000
	Mass	8454.38		0.000000	0.000000	0.000000
	Size	226		0.000000	0.000000	0.000000
Language pSTG (L) (−57,−47,15)-Salience SMG (L) (−60,−39,31)			16.70	0.000000	0.000000	
Language IFG (L) (−51,26,2)-Salience AInsula (L) (−44,13,1)			13.72	0.000000	0.000000	
Salience AInsula (R) (47,14,0)-Language IFG (R) (54,28,1)			13.16	0.000000	0.000000	
FrontoParietal PPC (R) (52,−52,45)-FrontoParietal LPFC (R) (41,38,30)			12.28	0.000000	0.000000	
Salience SMG (R) (62,−35,32)-Language pSTG (R) (59,−42,13)			11.04	0.000000	0.000000	
Salience RPFC (R) (32,46,27)-FrontoParietal LPFC (R) (41,38,30)			10.89	0.000000	0.000000	
Salience AInsula (L) (−44,13,1)-Salience AInsula (R) (47,14,0)			10.19	0.000000	0.000000	
Grey Matter—Language pSTG (R) (59,−42,13)			9.81	0.000000	0.000000	
Language pSTG (L) (−57,−47,15)-Language IFG (L) (−51,26,2)			9.68	0.000000	0.000000	
FrontoParietal LPFC (L) (−43,33,28)-FrontoParietal PPC (L) (−46,58,49)			9.56	0.000000	0.000000	
Salience AInsula (L) (−44,13,1)-Salience ACC (0,22,35)			9.20	0.000000	0.000000	
Salience RPFC (R) (32,46,27)-Salience AInsula (R) (47,14,0)			8.54	0.000000	0.000000	
Salience AInsula (R) (47,14,0)-Salience SMG (R) (62,−35,32)			8.45	0.000000	0.000000	
Salience RPFC (L) (−32,45,27)-Salience RPFC (R) (32,46,27)			8.44	0.000000	0.000000	
Salience ACC (0,22,35)—Salience RPFC (R) (32,46,27)			8.40	0.000000	0.000000	
Language IFG (R) (54,28,1)-Language pSTG (R) (59,−42,13)			8.32	0.000000	0.000000	
Salience ACC (0,22,35)—Salience AInsula (R) (47,14,0)			8.18	0.000000	0.000000	
FrontoParietal PPC (L) (−46,−58,49)-FrontoParietal.PPC (R) (52,−52,45)			7.92	0.000000	0.000000	
Salience.SMG (L) (−60,−39,31)-Salience.AInsula (L) (−44,13,1)			7.86	0.000000	0.000001	
DorsalAttention.IPS (R) (39,−42,54)-Salience.SMG (R) (62,−35,32)			7.85	0.000000	0.000001	
FrontoParietal.PPC (L) (−46,−58,49)-Language.pSTG (L) (−57,−47,15)			7.82	0.000000	0.000001	
SensoriMotor.Lateral (R) (56,−10,29)-Salience.AInsula (R) (47,14,0)			7.77	0.000000	0.000001	
Salience.SMG (L) (−60,−39,31)-Salience.AInsula (R) (47,14,0)			7.51	0.000000	0.000001	
Grey Matter—Language.pSTG (L) (−57,−47,15)			7.47	0.000000	0.000001	
FrontoParietal.PPC (L) (−46,−58,49)-Grey Matter			7.47	0.000000	0.000001	
Language.pSTG (L) (−57,−47,15)-Language.pSTG (R) (59,−42,13)			7.42	0.000000	0.000001	
FrontoParietal.LPFC (L) (−43,33,28)-Language.IFG (L) (−51,26,2)			7.37	0.000000	0.000001	
Salience.AInsula (L) (−44,13,1)-Salience.RPFC (L) (−32,45,27)			7.33	0.000000	0.000001	
Language.pSTG (R) (59,−42,13)-FrontoParietal.PPC (R) (52,−52,45)			7.26	0.000000	0.000002	
Salience.RPFC (L) (−32,45,27)-Salience.ACC (0,22,35)			7.14	0.000000	0.000002	
Salience.SMG (R) (62,−35,32)-FrontoParietal.PPC (R) (52,−52,45)			7.12	0.000000	0.000002	
Salience.RPFC (R) (32,46,27)-Salience.SMG (R) (62,−35,32)			6.86	0.000000	0.000004	
Salience.SMG (R) (62,−35,32)-Language.IFG (R) (54,28,1)			6.86	0.000000	0.000004	
Language.IFG (L) (−51,26,2)-Salience.SMG (L) (−60,−39,31)			6.84	0.000000	0.000004	
DorsalAttentionIPS (R) (39,−42,54)-SensoriMotorLateral (R) (56,−10,29)			6.70	0.000001	0.000005	
DorsalAttention.IPS (L) (−39,−43,52)-Salience.SMG (L) (−60,−39,31)			6.69	0.000001	0.000005	
Salience.AInsula (L) (−44,13,1)-Language.IFG (R) (54,28,1)			6.52	0.000001	0.000007	
Salience.SMG (L) (−60,−39,31)-Salience.SMG (R) (62,−35,32)			6.21	0.000002	0.000014	
Salience.AInsula (L) (−44,13,1)-Salience.SMG (R) (62,−35,32)			6.10	0.000002	0.000018	
Salience.RPFC (L) (−32,45,27)-Salience.AInsula (R) (47,14,0)			6.10	0.000002	0.000018	
FrontoParietal.LPFC (L) (−43,33,28)-Salience.RPFC (L) (−32,45,27)			6.09	0.000002	0.000018	
Language.IFG (R) (54,28,1)-FrontoParietal.LPFC (R) (41,38,30)			6.00	0.000003	0.000022	
Grey Matter—SensoriMotor.Lateral (R) (56,−10,29)			5.98	0.000003	0.000023	
Language.pSTG (L) (−57,−47,15)-Salience.AInsula (L) (−44,13,1)			5.66	0.000007	0.000051	
Salience.SMG (L) (−60,−39,31)-Language.IFG (R) (54,28,1)			5.55	0.000009	0.000064	
Grey Matter—FrontoParietal.PPC (R) (52,−52,45)			5.49	0.000011	0.000073	
Language.IFG (L) (−51,26,2)-Language.IFG (R) (54,28,1)			5.40	0.000013	0.000087	
SensoriMotor.Lateral (L) (−55,−12,29)-Salience.AInsula (L) (−44,13,1)			5.39	0.000014	0.000089	
DorsalAttention.IPS (R) (39,−42,54)-FrontoParietal.PPC(R) (52,−52,45)			5.25	0.000019	0.000121	
DorsalAttention.IPS (L) (−39,−43,52)-SensoriMotor.Lateral (L) (−55,−12,29)			5.25	0.000020	0.000121	
Salience.RPFC (R) (32,46,27)-Language.IFG (R) (54,28,1)			5.08	0.000030	0.000177	
Language.IFG (R) (54,28,1)-FrontoParietal.PPC (R) (52,−52,45)			4.96	0.000042	0.000235	
SensoriMotor.Lateral (R) (56,−10,29)-Salience.SMG (R) (62,−35,32)			4.92	0.000046	0.000257	
Salience.AInsula (L) (−44,13,1)-SensoriMotor.Lateral (R) (56,−10,29)			4.90	0.000049	0.000267	
Grey Matter—Language.IFG (R) (54,28,1)			4.87	0.000052	0.000282	
SensoriMotor.Lateral (L) (−55,−12,29)-Grey Matter			4.83	0.000058	0.000310	
Language.pSTG (L) (−57,−47,15)-Language.IFG (R) (54,28,1)			4.77	0.000068	0.000360	
Salience.ACC (0,22,35)—Salience.SMG (R) (62,−35,32)			4.74	0.000073	0.000377	
FrontoParietal.PPC (L) (−46,−58,49)-Salience.SMG (L) (−60,−39,31)			4.73	0.000074	0.000377	
FrontoParietal.PPC (L) (−46,−58,49)-Language.IFG (L) (−51,26,2)			4.72	0.000077	0.000388	
Language.IFG (L) (−51,26,2)-Salience.RPFC (L) (−32,45,27)			4.55	0.000121	0.000576	
Salience.AInsula (R) (47,14,0)-Language.pSTG (R) (59,−42,13)			4.41	0.000173	0.000785	
DorsalAttention.IPS (L) (−39,−43,52)-FrontoParietal.PPC (L) (−46,−58,49)			4.26	0.000256	0.001095	
Salience.RPFC (R) (32,46,27)-FrontoParietal.PPC (R) (52,−52,45)			4.25	0.000257	0.001095	
SensoriMotor.Lateral (L) (−55,−12,29)-Salience.SMG (L) (−60,−39,31)			4.22	0.000281	0.001186	
Grey Matter—Salience.SMG (R) (62,−35,32)			4.20	0.000293	0.001229	
Salience.SMG (R) (62,−35,32)-FrontoParietal.LPFC (R) (41,38,30)			4.07	0.000419	0.001715	
DorsalAttention.IPS (R) (39,−42,54)-Salience.AInsula (R) (47,14,0)			4.04	0.000449	0.001823	
SensoriMotor.Lateral (L) (−55,−12,29)-FrontoParietal.LPFC (L) (−43,33,28)			3.97	0.000541	0.002134	
Salience.AInsula (L) (−44,13,1)-Salience.RPFC (R) (32,46,27)			3.97	0.000542	0.002134	
SensoriMotor.Lateral (R) (56,−10,29)-Language.IFG (R) (54,28,1)			3.96	0.000543	0.002134	
DorsalAttention.IPS (R) (39,−42,54)-FrontoParietal.LPFC (R) (41,38,30)			3.92	0.000613	0.002312	
Grey Matter—Language.IFG (L) (−51,26,2)			3.91	0.000618	0.002315	
Salience.RPFC (R) (32,46,27)-Language.pSTG (R) (59,−42,13)			3.88	0.000677	0.002496	
SensoriMotor.Lateral (R) (56,−10,29)-FrontoParietal.PPC (R) (52,−52,45)			3.88	0.000681	0.002496	
Grey Matter—Salience.SMG (L) (−60,−39,31)			3.83	0.000766	0.002769	
Salience.SMG (L) (−60,−39,31)-Salience.RPFC (L) (−32,45,27)			3.78	0.000859	0.003066	
Grey Matter—FrontoParietal.LPFC (R) (41,38,30)			3.75	0.000930	0.003296	
Language.IFG (L) (−51,26,2)-Language.pSTG (R) (59,−42,13)			3.68	0.001112	0.003913	
Salience.ACC (0,22,35)—SensoriMotor.Lateral (R) (56,−10,29)			3.65	0.001213	0.004227	
DorsalAttention.IPS (L) (−39,−43,52)-Salience.AInsula (L) (−44,13,1)			3.65	0.001217	0.004227	
Language.IFG (L) (−51,26,2)-Salience.AInsula (R) (47,14,0)			3.59	0.001390	0.004736	
Salience.AInsula (R) (47,14,0)-FrontoParietal.LPFC (R) (41,38,30)			3.59	0.001407	0.004763	
Grey Matter—Salience.AInsula (R) (47,14,0)			3.38	0.002369	0.007357	
Grey Matter—DorsalAttention.IPS (R) (39,−42,54)			3.22	0.003542	0.010276	
SensoriMotor.Lateral (R) (56,−10,29)-FrontoParietal.LPFC (R) (41,38,30)			3.21	0.003588	0.010353	
Salience.RPFC (L) (−32,45,27)-Salience.SMG (R) (62,−35,32)			3.14	0.004253	0.011944	
Grey Matter—Salience.AInsula (L) (−44,13,1)			3.11	0.004589	0.012667	
Salience.SMG (L) (−60,−39,31)-Language.pSTG (R) (59,−42,13)			3.08	0.004972	0.013394	
Salience.AInsula (L) (−44,13,1)-Language.pSTG (R) (59,−42,13)			3.07	0.005150	0.013733	
Language.pSTG (L) (−57,−47,15)-Salience.AInsula (R) (47,14,0)			2.96	0.006682	0.017295	
Salience.AInsula (L) (−44,13,1)-DorsalAttention.IPS (R) (39,−42,54)			2.90	0.007595	0.019373	
SensoriMotor.Lateral (L) (−55,−12,29)-Language.IFG (L) (−51,26,2)			2.84	0.008857	0.021956	
FrontoParietal.LPFC (L) (−43,33,28)-Salience.AInsula (L) (−44,13,1)			2.81	0.009504	0.023125	
DorsalAttention.IPS(L) (−39,−43,52)-FrontoParietal.LPFC (L) (−43,33,28)			2.79	0.009868	0.023901	
DorsalAttention.FEF (L) (−27,−9,64)-DorsalAttention.IPS (L) (−39,−43,52)			2.67	0.013260	0.030708	
Salience.ACC (0,22,35)—Language.IFG (R) (54,28,1)			2.65	0.013653	0.031479	
FrontoParietal.LPFC (L) (−43,33,28)-Language.pSTG (L) (−57,−47,15)			2.61	0.015160	0.034627	
Salience.RPFC (R) (32,46,27)-DorsalAttention.IPS (R) (39,−42,54)			2.59	0.015644	0.035299	
DorsalAttention.IPS (L) (−39,−43,52)-Salience.RPFC (L) (−32,45,27)			2.52	0.018576	0.041039	
SensoriMotor.Lateral (L) (−55,−12,29)-Salience.RPFC (L) (−32,45,27)			2.37	0.025557	0.054632	
Salience.SMG (L) (−60,−39,31)-Salience.RPFC (R) (32,46,27)			2.37	0.025732	0.054785	
DorsalAttention.IPS (R) (39,−42,54)-Language.IFG (R) (54,28,1)			2.37	0.026029	0.055122	
DorsalAttention.FEF (L) (−27,−9,64)-Salience.RPFC (L) (−32,45,27)			2.36	0.026369	0.055249	
SensoriMotor.Lateral (L) (−55,−12,29)-Language.pSTG (L) (−57,−47,15)			2.32	0.028845	0.059495	
Salience.SMG (L) (−60,−39,31)-Salience.ACC (0,22,35)			2.30	0.029968	0.061329	
Language.pSTG (L) (−57,−47,15)-Salience.SMG (R) (62,−35,32)			2.27	0.032417	0.065081	
FrontoParietal.LPFC (L) (−43,33,28)-Grey Matter			2.25	0.033526	0.066614	
Language.pSTG (R) (59,−42,13)-FrontoParietal.LPFC (R) (41,38,30)			2.24	0.034150	0.067334	
DorsalAttention.FEF (L) (−27,−9,64)-Salience.ACC (0,22,35)			2.24	0.034177	0.067334	
FrontoParietal.LPFC (L) (−43,33,28)-Salience.SMG (L) (−60,−39,31)			2.16	0.040530	0.078101	
Salience.AInsula (R) (47,14,0)-FrontoParietal.PPC (R) (52,−52,45)			2.10	0.045912	0.086268	
Salience.RPFC (R) (32,46,27)-SensoriMotor.Lateral (R) (56,−10,29)			2.10	0.046229	0.086556	
Cluster 3/34						
	Score	355.71		0.000000	0.000000	0.000000
	Mass	916.48		0.000000	0.000000	0.000000
	Size	26		0.000000	0.000000	0.000000
DefaultMode.LP (R) (47,−67,29)-Grey Matter			8.77	0.000000	0.000000	
DefaultMode.LP (L) (−39,−77,33)-Grey Matter			8.43	0.000000	0.000000	
DefaultMode.LP (L) (−39,−77,33)-FrontoParietal.PPC (L) (−46,−58,49)			7.41	0.000000	0.000001	
DefaultMode.PCC (1,−61,38)-Grey Matter			7.00	0.000000	0.000003	
Visual.Lateral (R) (38,−72,13)-Grey Matter			6.59	0.000001	0.000006	
Visual.Medial (2,−79,12)-Grey Matter			6.15	0.000002	0.000016	
DefaultMode.MPFC (1,55,−3)-Grey Matter			5.69	0.000006	0.000047	
Visual.Lateral (L) (−37,−79,10)-Language.pSTG (L) (−57,−47,15)			4.75	0.000072	0.000376	
Visual.Lateral (L) (−37,−79,10)-Grey Matter			4.74	0.000074	0.000377	
DefaultMode.LP (L) (−39,−77,33)-Language.pSTG (L) (−57,−47,15)			4.62	0.000101	0.000490	
DefaultMode.MPFC (1,55,−3)-Language.IFG (L) (−51,26,2)			3.61	0.001322	0.004534	
DefaultMode.MPFC (1,55,−3)-Language.pSTG (L) (−57,−47,15)			2.79	0.009933	0.023904	
Visual.Lateral (L) (−37,−79,10)-Language.IFG (L) (−51,26,2)			2.36	0.026219	0.055155	
Cluster 4/34						
	Score	188.22		0.000000	0.000000	0.000000
	Mass	396.83		0.000000	0.000000	0.000000
	Size	14		0.002785	0.011835	0.055000
DefaultMode.LP (R) (47,−67,29)-Language.pSTG (R) (59,−42,13)			8.53	0.000000	0.000000	
DefaultMode.LP (R) (47,−67,29)-FrontoParietal.PPC (R) (52,−52,45)			8.39	0.000000	0.000000	
DefaultMode.LP (R) (47,−67,29)-FrontoParietal.LPFC (R) (41,38,30)			4.40	0.000175	0.000785	
DefaultMode.MPFC (1,55,−3)-Language.pSTG (R) (59,−42,13)			3.91	0.000626	0.002329	
DefaultMode.PCC (1,−61,38)-Language.pSTG (R) (59,−42,13)			3.15	0.004203	0.011867	
DefaultMode.LP (R) (47,−67,29)-Salience.SMG (R) (62,−35,32)			2.32	0.028569	0.059387	
DefaultMode.MPFC (1,55,−3)-Language.IFG (R) (54,28,1)			2.31	0.029582	0.060775	
Cluster 5/34						
	Score	125.54		0.000000	0.000000	0.000000
	Mass	384.25		0.000000	0.000000	0.000000
	Size	20		0.000349	0.001979	0.008000
DefaultMode.PCC (1,−61,38)-SensoriMotor.Lateral (R) (56,−10,29)			−8.41	0.000000	0.000000	
DefaultMode.PCC (1,−61,38)-Salience.AInsula (R) (47,14,0)			−5.11	0.000028	0.000170	
DefaultMode.LP (L) (−39,−77,33)-Salience.AInsula (R) (47,14,0)			−4.97	0.000040	0.000229	
DefaultMode.PCC (1,−61,38)-Language.IFG (R) (54,28,1)			−4.46	0.000150	0.000694	
DefaultMode.LP (L) (−39,−77,33)-Salience.SMG (R) (62,−35,32)			−3.56	0.001514	0.004995	
DefaultMode.LP (L) (−39,−77,33)-Language.IFG (R) (54,28,1)			−3.24	0.003374	0.009898	
DefaultMode.LP (L) (−39,−77,33)-SensoriMotor.Lateral (R) (56,−10,29)			−3.08	0.004954	0.013394	
DefaultMode.LP (R) (47,−67,29)-Salience.AInsula (R) (47,14,0)			−2.74	0.011175	0.026340	
DefaultMode.MPFC (1,55,−3)-Salience.SMG (R) (62,−35,32)			−2.49	0.019981	0.043775	
DefaultMode.PCC (1,−61,38)-Salience.SMG (R) (62,−35,32)			−2.08	0.048206	0.089309	
Cluster 6/34						
	Score	118.81		0.000000	0.000000	0.000000
	Mass	853.83		0.000000	0.000000	0.000000
	Size	70		0.000000	0.000000	0.000000
DefaultMode.LP (R) (47,−67,29)-Salience.RPFC (L) (−32,45,27)			−6.32	0.000001	0.000011	
Cerebellar.Anterior (0,−63,−30)-Salience.ACC (0,22,35)			−5.51	0.000010	0.000069	
DefaultMode.LP (R) (47,−67,29)-Salience.AInsula (L) (−44,13,1)			−4.66	0.000089	0.000445	
DefaultMode.PCC (1,−61,38)-Salience.AInsula (L) (−44,13,1)			−4.65	0.000092	0.000453	
Visual.Lateral (R) (38,−72,13)-Salience.RPFC (L) (−32,45,27)			−4.47	0.000148	0.000692	
DefaultMode.LP (R) (47,−67,29)-Salience.SMG (L) (−60,−39,31)			−4.38	0.000186	0.000820	
DefaultMode.PCC (1,−61,38)-Language.IFG (L) (−51,26,2)			−4.29	0.000235	0.001016	
Visual.Lateral (R) (38,−72,13)-Salience.ACC (0,22,35)			−4.14	0.000344	0.001430	
DefaultMode.LP (R) (47,−67,29)-Salience.ACC (0,22,35)			−3.96	0.000546	0.002134	
Visual.Lateral (L) (−37,−79,10)-Salience.ACC (0,22,35)			−3.87	0.000688	0.002507	
Visual.Lateral (L) (−37,−79,10)-Salience.RPFC (L) (−32,45,27)			−3.63	0.001272	0.004389	
Visual.Lateral (L) (−37,−79,10)-SensoriMotor.Lateral (R) (56,−10,29)			−3.56	0.001525	0.005000	
DefaultMode.LP (L) (−39,−77,33)-Salience.RPFC (R) (32,46,27)			−3.45	0.001983	0.006271	
DefaultMode.LP (L) (−39,−77,33)-Salience.AInsula (L) (−44,13,1)			−3.44	0.002057	0.006465	
Visual.Occipital (0,−93,−4)-SensoriMotor.Lateral (R) (56,−10,29)			−3.32	0.002745	0.008331	
Visual.Occipital (0,−93,−4)-Salience.ACC (0,22,35)			−3.29	0.002947	0.008790	
Visual.Occipital (0,−93,−4)-Salience.SMG (R) (62,−35,32)			−3.22	0.003525	0.010276	
Visual.Occipital (0,−93,−4)-Salience.RPFC (R) (32,46,27)			−3.12	0.004528	0.012611	
DefaultMode.MPFC (1,55,−3)-Salience.RPFC (L) (−32,45,27)			−3.11	0.004606	0.012667	
Visual.Lateral (L) (−37,−79,10)-Salience.SMG (R) (62,−35,32)			−2.94	0.006940	0.017788	
Cerebellar.Posterior (0,−79,−32)-Salience.ACC (0,22,35)			−2.82	0.009323	0.022941	
DefaultMode.LP (L) (−39,−77,33)-Salience.ACC (0,22,35)			−2.81	0.009385	0.022941	
DefaultMode.PCC (1,−61,38)-Salience.SMG (L) (−60,−39,31)			−2.79	0.009960	0.023904	
Visual.Lateral (R) (38,−72,13)-Salience.SMG (L) (−60,−39,31)			−2.79	0.010011	0.023919	
Visual.Medial (2,−79,12)-Salience.ACC (0,22,35)			−2.77	0.010344	0.024491	
Visual.Lateral (L) (−37,−79,10)-Salience.RPFC (R) (32,46,27)			−2.68	0.012817	0.029812	
Visual.Medial (2,−79,12)-Salience.SMG (R) (62,−35,32)			−2.63	0.014391	0.033036	
Cerebellar.Anterior (0,−63,−30)-DorsalAttention.IPS (R) (39,−42,54)			−2.57	0.016571	0.036992	
DefaultMode.LP (L) (−39,−77,33)-Salience.RPFC (L) (−32,45,27)			−2.57	0.016604	0.036992	
Visual.Lateral (R) (38,−72,13)-Salience.RPFC (R) (32,46,27)			−2.45	0.021611	0.046956	
DefaultMode.LP (R) (47,−67,29)-Language.IFG (L) (−51,26,2)			−2.38	0.025499	0.054632	
Cerebellar.Posterior (0,−79,−32)-SensoriMotor.Lateral (R) (56,−10,29)			−2.29	0.031057	0.063314	
Visual.Occipital (0,−93,−4)-Salience.AInsula (R) (47,14,0)			−2.27	0.031800	0.064567	
Visual.Medial (2,−79,12)-DorsalAttention.IPS (R) (39,−42,54)			−2.23	0.034611	0.067612	
Cerebellar.Posterior (0,−79,−32)-DorsalAttention.IPS (R) (39,−42,54)			−2.12	0.044535	0.084890	
Cluster 7/34						
	Score	90.26		0.000000	0.000000	0.000000
	Mass	439.25		0.000000	0.000000	0.000000
	Size	34		0.000000	0.000000	0.000000
SensoriMotor.Superior (0,−31,67)-FrontoParietal.LPFC (R) (41,38,30)			−5.42	0.000012	0.000085	
SensoriMotor.Lateral (L) (−55,−12,29)-FrontoParietal.LPFC (R) (41,38,30)			−5.42	0.000013	0.000085	
DorsalAttention.FEF (L) (−27,−9,64)-FrontoParietal.PPC (R) (52,−52,45)			−4.61	0.000101	0.000490	
DorsalAttention.FEF (L) (−27,−9,64)-FrontoParietal.LPFC (R) (41,38,30)			−4.45	0.000156	0.000718	
FrontoParietal.LPFC (L) (−43,33,28)-Language.pSTG (R) (59,−42,13)			−4.40	0.000174	0.000785	
SensoriMotor.Lateral (L) (−55,−12,29)-Language.pSTG (R) (59,−42,13)			−3.46	0.001953	0.006212	
FrontoParietal.LPFC (L) (−43,33,28)-Salience.SMG (R) (62,−35,32)			−3.40	0.002248	0.007023	
DorsalAttention.IPS (L) (−39,−43,52)-Language.pSTG (R) (59,−42,13)			−3.30	0.002898	0.008714	
SensoriMotor.Lateral (L) (−55,−12,29)-FrontoParietal.PPC (R) (52,−52,45)			−3.17	0.003953	0.011221	
SensoriMotor.Superior (0,−31,67)-Language.IFG (R) (54,28,1)			−3.07	0.005103	0.013676	
SensoriMotor.Superior (0,−31,67)-Salience.AInsula (R) (47,14,0)			−2.99	0.006113	0.015980	
FrontoParietal.LPFC (L) (−43,33,28)-Language.IFG (R) (54,28,1)			−2.78	0.010188	0.024231	
SensoriMotor.Lateral (L) (−55,−12,29)-Language.IFG (R) (54,28,1)			−2.73	0.011524	0.027042	
DorsalAttention.FEF (L) (−27,−9,64)-Salience.SMG (R) (62,−35,32)			−2.71	0.011970	0.027965	
DorsalAttention.FEF (L) (−27,−9,64)-Language.pSTG (R) (59,−42,13)			−2.27	0.031917	0.064567	
DorsalAttention.IPS (L) (−39,−43,52)-FrontoParietal.LPFC (R) (41,38,30)			−2.25	0.033559	0.066614	
DorsalAttention.IPS (L) (−39,−43,52)-FrontoParietal.PPC (R) (52,−52,45)			−2.17	0.039383	0.076421	
Cluster 8/34						
	Score	78.54		0.000061	0.000208	0.001000
	Mass	199.95		0.000055	0.000232	0.001000
	Size	12		0.005748	0.019542	0.106000
DefaultMode.LP (R) (47,−67,29)-SensoriMotor.Lateral (L) (−55,−12,29)			−6.24	0.000002	0.000014	
DefaultMode.PCC (1,−61,38)-SensoriMotor.Lateral (L) (−55,−12,29)			−5.04	0.000033	0.000193	
DefaultMode.LP (R) (47,−67,29)-DorsalAttention.IPS (L) (−39,−43,52)			−3.79	0.000851	0.003057	
DefaultMode.LP (R) (47,−67,29)-FrontoParietal.LPFC (L) (−43,33,28)			−3.25	0.003298	0.009729	
DefaultMode.MPFC (1,55,−3)-DorsalAttention.IPS (L) (−39,−43,52)			−2.40	0.024261	0.052285	
DefaultMode.LP (R) (47,−67,29)-DorsalAttention.FEF (L) (−27,−9,64)			−2.23	0.034702	0.067612	
DefaultMode.LP (R) (47,−67,29)-SensoriMotor.Lateral (L) (−55,−12,29)			−6.24	0.000002	0.000014	
DefaultMode.PCC (1,−61,38)-SensoriMotor.Lateral (L) (−55,−12,29)			−5.04	0.000033	0.000193	
DefaultMode.LP (R) (47,−67,29)-DorsalAttention.IPS (L) (−39,−43,52)			−3.79	0.000851	0.003057	
DefaultMode.LP (R) (47,−67,29)-FrontoParietal.LPFC (L) (−43,33,28)			−3.25	0.003298	0.009729	
DefaultMode.MPFC (1,55,−3)-DorsalAttention.IPS (L) (−39,−43,52)			−2.40	0.024261	0.052285	
DefaultMode.LP (R) (47,−67,29)-DorsalAttention.FEF (L) (−27,−9,64)			−2.23	0.034702	0.067612	
Cluster 9/34						
	Score	143.43		0.000000	0.000000	0.000000
	Mass	188.19		0.000062	0.000232	0.001000
	Size	6		0.063172	0.113045	0.675000
SensoriMotor.Lateral (L) (−55,−12,29)-SensoriMotor.Lateral (R)(56,−10,29)			7.58	0.000000	0.000001	
DorsalAttention.IPS (L) (−39,−43,52)-DorsalAttention.IPS (R) (39,−42,54)			4.61	0.000104	0.000498	
SensoriMotor.Lateral (L) (−55,−12,29)-Salience.AInsula (R) (47,14,0)			3.92	0.000605	0.002300	
Cluster 10/34						
	Score	55.03		0.000438	0.001241	0.009000
	Mass	174.82		0.000069	0.000232	0.001000
	Size	14		0.002785	0.011835	0.055000
Visual.Medial (2,−79,12)-DorsalAttention.IPS (L) (−39,−43,52)			−5.70	0.000006	0.000046	
Visual.Lateral (L) (−37,−79,10)-SensoriMotor.Superior (0,−31,67)			−3.51	0.001718	0.005572	
Visual.Medial (2,−79,12)-SensoriMotor.Superior (0,−31,67)			−3.48	0.001877	0.006007	
Visual.Occipital (0,−93,−4)-DorsalAttention.IPS (L) (−39,−43,52)			−3.12	0.004538	0.012611	
Visual.Lateral (R) (38,−72,13)-DorsalAttention.FEF (L) (−27,−9,64)			−2.87	0.008253	0.020749	
Visual.Medial (2,−79,12)-DorsalAttention.FEF (L) (−27,−9,64)			−2.84	0.008755	0.021909	
Visual.Lateral (R) (38,−72,13)-SensoriMotor.Superior (0,−31,67)			−2.10	0.045692	0.086233	
Cluster 11/34						
	Score	117.49		0.000000	0.000000	0.000000
	Mass	164.76		0.000075	0.000232	0.001000
	Size	6		0.063172	0.113045	0.675000
Visual.Lateral (R) (38,−72,13)-SensoriMotor.Lateral (L) (−55,−12,29)			−5.55	0.000009	0.000064	
Visual.Lateral (R) (38,−72,13)-FrontoParietal.LPFC (L) (−43,33,28)			−5.37	0.000014	0.000092	
Visual.Lateral (R) (38,−72,13)-FrontoParietal.PPC (L) (−46,−58,49)			−4.78	0.000067	0.000355	
Cluster 12/34						
	Score	37.05		0.003830	0.010018	0.079000
	Mass	121.17		0.001439	0.004076	0.028000
	Size	12		0.005748	0.019542	0.106000
Cerebellar.Anterior (0,−63,−30)-Grey Matter			4.39	0.000183	0.000814	
Cerebellar.Posterior (0,−79,−32)-Language.IFG (L) (−51,26,2)			3.57	0.001489	0.004944	
Visual.Occipital (0,−93,−4)-Language.IFG (L) (−51,26,2)			3.10	0.004738	0.012962	
Cerebellar.Anterior (0,−63,−30)-Salience.SMG (L) (−60,−39,31)			2.82	0.009367	0.022941	
Cerebellar.Anterior (0,−63,−30)-Language.pSTG (L) (−57,−47,15)			2.37	0.026100	0.055122	
Cerebellar.Posterior (0,−79,−32)-Language.pSTG (L) (−57,−47,15)			2.34	0.027347	0.057073	

**Table A2:** Statistical Values of ROI-to-ROI Connectivity Analysis for Handball players.

Analysis Unit			T (12)−Value	p-unc	p-FDR	*p*-FWE
Cluster 1/40						
	Mass	3177.80		0.000000	0.000000	0.000000
DefaultMode.LP (R) (47,−67,29)-DefaultMode.LP (L) (−39,−77,33)			9.88	0.000000	0.000047	
Salience.ACC (0,22,35)—Salience.AInsula (L) (−44,13,1)			8.70	0.000002	0.000139	
Salience.AInsula (R) (47,14,0)-Language.IFG (R) (54,28,1)			8.40	0.000002	0.000171	
Salience.SMG (R) (62,−35,32)-Language.pSTG (R) (59,−42,13)			7.58	0.000007	0.000372	
FrontoParietal.LPFC (R) (41,38,30)-FrontoParietal.PPC (R) (52,−52,45)			7.52	0.000007	0.000372	
FrontoParietal.PPC (R) (52,−52,45)-Language.pSTG (R) (59,−42,13)			6.61	0.000025	0.000865	
DefaultMode.LP (R) (47,−67,29)-DefaultMode.PCC (1,−61,38)			6.59	0.000026	0.000865	
Language.pSTG (R) (59,−42,13)-Grey Matter			6.58	0.000026	0.000865	
Language.pSTG (R) (59,−42,13)-DefaultMode.LP (R) (47,−67,29)			6.47	0.000031	0.000958	
SensoriMotor.Lateral (R) (56,−10,29)-DorsalAttention.IPS (R) (39,−42,54)			6.40	0.000034	0.000963	
Grey Matter—DefaultMode.LP (L) (−39,−77,33)			6.36	0.000036	0.000963	
Salience.SMG (R) (62,−35,32)-DorsalAttention.IPS (R) (39,−42,54)			6.35	0.000036	0.000963	
FrontoParietal.PPC (R) (52,−52,45)-DefaultMode.LP (R) (47,−67,29)			6.28	0.000041	0.001025	
Grey Matter—DefaultMode.LP (R) (47,−67,29)			6.19	0.000047	0.001117	
Salience.SMG (R) (62,−35,32)-FrontoParietal.PPC (R) (52,−52,45)			6.03	0.000059	0.001356	
Salience.AInsula (L) (−44,13,1)-Salience.AInsula (R) (47,14,0)			5.90	0.000072	0.001525	
FrontoParietal.PPC (R) (52,−52,45)-Grey Matter			5.55	0.000126	0.002384	
Salience.AInsula (R) (47,14,0)-SensoriMotor.Lateral (R) (56,−10,29)			5.36	0.000170	0.002684	
Salience.SMG (L) (−60,−39,31)-Salience.AInsula (R) (47,14,0)			5.35	0.000174	0.002684	
Grey Matter—DefaultMode.PCC (1,−61,38)			5.34	0.000177	0.002684	
FrontoParietal.LPFC (R) (41,38,30)-Grey Matter			5.15	0.000243	0.002916	
Salience.RPFC (R) (32,46,27)-Salience.RPFC (L) (−32,45,27)			4.98	0.000318	0.003576	
DefaultMode.LP (L) (−39,−77,33)-DefaultMode.MPFC (1,55,−3)			4.84	0.000404	0.004311	
Salience.ACC (0,22,35)—Salience.AInsula (R) (47,14,0)			4.84	0.000408	0.004311	
Language.IFG (R) (54,28,1)-Salience.SMG (R) (62,−35,32)			4.72	0.000498	0.005156	
Salience.AInsula (L) (−44,13,1)-Language.IFG (R) (54,28,1)			4.69	0.000521	0.005291	
Grey Matter—DefaultMode.MPFC (1,55,−3)			4.68	0.000534	0.005321	
Language.IFG (R) (54,28,1)-FrontoParietal.LPFC (R) (41,38,30)			4.60	0.000610	0.005901	
Salience.SMG (R) (62,−35,32)-SensoriMotor.Lateral (R) (56,−10,29)			4.60	0.000615	0.005901	
Salience.ACC (0,22,35)—Salience.RPFC (L) (−32,45,27)			4.56	0.000656	0.006183	
Salience.RPFC (R) (32,46,27)-Salience.ACC (0,22,35)			4.43	0.000827	0.007526	
Salience.SMG (L) (−60,−39,31)-Salience.AInsula (L) (−44,13,1)			4.39	0.000874	0.007693	
Salience.SMG (R) (62,−35,32)-FrontoParietal.LPFC (R) (41,38,30)			4.33	0.000974	0.008159	
Language.IFG (R) (54,28,1)-Grey Matter			4.30	0.001025	0.008378	
Salience.AInsula (L) (−44,13,1)-Salience.SMG (R) (62,−35,32)			4.30	0.001031	0.008378	
Salience.SMG (L) (−60,−39,31)-Language.IFG (R) (54,28,1)			4.27	0.001085	0.008684	
Salience.AInsula (R) (47,14,0)-Salience.SMG (R) (62,−35,32)			4.26	0.001112	0.008766	
Salience.RPFC (R) (32,46,27)-Salience.AInsula (R) (47,14,0)			4.19	0.001247	0.009541	
DefaultMode.PCC (1,−61,38)-DefaultMode.MPFC (1,55,−3)			4.03	0.001675	0.011973	
Language.IFG (R) (54,28,1)-Language.pSTG (R) (59,−42,13)			4.03	0.001678	0.011973	
SensoriMotor.Lateral (R) (56,−10,29)-Grey Matter			3.91	0.002058	0.013662	
Salience.RPFC (L) (−32,45,27)-Salience.AInsula (L) (−44,13,1)			3.91	0.002070	0.013662	
Salience.SMG (L) (−60,−39,31)-Salience.SMG (R) (62,−35,32)			3.81	0.002483	0.016161	
DorsalAttention.IPS (R) (39,−42,54)-FrontoParietal.PPC (R) (52,−52,45)			3.80	0.002510	0.016161	
Salience.AInsula (L) (−44,13,1)-SensoriMotor.Lateral (R) (56,−10,29)			3.76	0.002716	0.016950	
DefaultMode.LP (L) (−39,−77,33)-DefaultMode.PCC (1,−61,38)			3.71	0.002955	0.017963	
Language.IFG (R) (54,28,1)-FrontoParietal.PPC (R) (52,−52,45)			3.65	0.003329	0.019315	
DefaultMode.LP (R) (47,−67,29)-DefaultMode.MPFC (1,55,−3)			3.64	0.003395	0.019482	
Salience.RPFC (R) (32,46,27)-Salience.SMG (R) (62,−35,32)			3.48	0.004539	0.023609	
DorsalAttention.IPS (R) (39,−42,54)-Grey Matter			3.45	0.004829	0.024755	
Language.IFG (R) (54,28,1)-SensoriMotor.Lateral (R) (56,−10,29)			3.36	0.005678	0.028281	
DorsalAttention.IPS (R) (39,−42,54)-FrontoParietal.LPFC (R) (41,38,30)			3.24	0.007055	0.032963	
FrontoParietal.LPFC (R) (41,38,30)-DefaultMode.LP (R) (47,−67,29)			3.01	0.010799	0.045223	
Salience.ACC (0,22,35)—Language.IFG (R) (54,28,1)			2.98	0.011432	0.046431	
Salience.RPFC (L) (−32,45,27)-Salience.AInsula (R) (47,14,0)			2.96	0.011839	0.047357	
Salience.RPFC (R) (32,46,27)-Language.IFG (R) (54,28,1)			2.93	0.012604	0.050035	
Salience.AInsula (R) (47,14,0)-DorsalAttention.IPS (R) (39,−42,54)			2.84	0.014821	0.056705	
SensoriMotor.Lateral (R) (56,−10,29)-FrontoParietal.PPC (R) (52,−52,45)			2.73	0.018135	0.065140	
Salience.SMG (R) (62,−35,32)-Grey Matter			2.57	0.024741	0.083871	
Salience.AInsula (L) (−44,13,1)-DorsalAttention.IPS (R) (39,−42,54)			2.50	0.027935	0.090488	
SensoriMotor.Lateral (R) (56,−10,29)-Language.pSTG (R) (59,−42,13)			2.48	0.028934	0.092032	
Salience.AInsula (R) (47,14,0)-FrontoParietal.LPFC (R) (41,38,30)			2.45	0.030374	0.095067	
Language.IFG (R) (54,28,1)-DorsalAttention.IPS (R) (39,−42,54)			2.40	0.033823	0.103229	
Salience.ACC (0,22,35)—Salience.SMG (L) (−60,−39,31)			2.38	0.034910	0.105329	
Salience.RPFC (R) (32,46,27)-Salience.AInsula (L) (−44,13,1)			2.34	0.037116	0.110718	
FrontoParietal.LPFC (R) (41,38,30)-Language.pSTG (R) (59,−42,13)			2.28	0.041394	0.121422	
Language.pSTG (R) (59,−42,13)-DefaultMode.MPFC (1,55,−3)			2.25	0.043840	0.127887	
Cluster 2/40						
	Mass	1038.87		0.000000	0.000000	0.000000
Visual.Medial (2,−79,12)- Visual.Lateral (R) (38,−72,13)			9.89	0.000000	0.000047	
Cerebellar.Posterior (0,−79,−32)-Cerebellar.Anterior (0,−63,−30)			9.81	0.000000	0.000047	
Visual.Occipital (0,−93,−4)-Visual.Medial (2,−79,12)			8.18	0.000003	0.000198	
Visual.Occipital (0,−93,−4)-Visual.Lateral (R) (38,−72,13)			7.42	0.000008	0.000387	
Cerebellar.Anterior (0,−63,−30)-Visual.Medial (2,−79,12)			5.99	0.000063	0.001387	
Visual.Lateral (L) (−37,−79,10)-Visual.Lateral (R) (38,−72,13)			5.72	0.000095	0.001865	
Visual.Occipital (0,−93,−4)-Visual.Lateral (L) (−37,−79,10)			5.34	0.000177	0.002684	
Visual.Medial (2,−79,12)-Visual.Lateral (L) (−37,−79,10)			5.33	0.000178	0.002684	
Cerebellar.Posterior (0,−79,−32)-Visual.Occipital (0,−93,−4)			4.48	0.000755	0.006990	
Cerebellar.Posterior (0,−79,−32)-Visual.Medial (2,−79,12)			4.23	0.001174	0.009117	
Cerebellar.Anterior (0,−63,−30)-Visual.Lateral (R) (38,−72,13)			3.93	0.002011	0.013662	
Cerebellar.Anterior (0,−63,−30)-Visual.Occipital (0,−93,−4)			3.66	0.003265	0.019315	
Cerebellar.Anterior (0,−63,−30)-Visual.Lateral (L) (−37,−79,10)			3.34	0.005868	0.028956	
Cluster 3/40						
	Mass	910.51		0.000000	0.000000	0.000000
Salience.SMG (L) (−60,−39,31)-Language.pSTG (L) (−57,−47,15)			13.91	0.000000	0.000005	
Salience.AInsula (L) (−44,13,1)-Language.IFG (L) (−51,26,2)			10.51	0.000000	0.000047	
Language.IFG (R) (54,28,1)-Language.pSTG (L) (−57,−47,15)			4.92	0.000353	0.003883	
Salience.SMG (L) (−60,−39,31)-Language.IFG (L) (−51,26,2)			4.37	0.000917	0.007940	
Salience.SMG (L) (−60,−39,31)-DorsalAttention.IPS (L) (−39,−43,52)			4.35	0.000944	0.008035	
Salience.SMG (L) (−60,−39,31)-FrontoParietal.PPC (L) (−46,−58,49)			3.76	0.002729	0.016950	
Language.IFG (R) (54,28,1)-Language.IFG (L) (−51,26,2)			3.61	0.003608	0.020051	
Salience.AInsula (L) (−44,13,1)-SensoriMotor.Lateral (L) (−55,−12,29)			3.52	0.004243	0.022862	
Salience.AInsula (R) (47,14,0)-SensoriMotor.Lateral (L) (−55,−12,29)			3.31	0.006229	0.030264	
Salience.SMG (L) (−60,−39,31)-SensoriMotor.Lateral (L) (−55,−12,29)			3.20	0.007671	0.035146	
Salience.RPFC (L) (−32,45,27)-FrontoParietal.LPFC (L) (−43,33,28)			3.05	0.010060	0.042494	
Salience.AInsula (L) (−44,13,1)-Language.pSTG (L) (−57,−47,15)			2.68	0.020155	0.070014	
Salience.RPFC (L) (−32,45,27)-Language.IFG (L) (−51,26,2)			2.48	0.028738	0.091961	
Salience.AInsula (R) (47,14,0)-Language.IFG (L) (−51,26,2)			2.43	0.031639	0.098253	
Cluster 4/40						
	Mass	371.40		0.000126	0.001262	0.002000
Salience.SMG (R) (62,−35,32)-DefaultMode.LP (L) (−39,−77,33)			−5.25	0.000204	0.002730	
SensoriMotor.Lateral (R) (56,−10,29)-DefaultMode.PCC (1,−61,38)			−5.24	0.000207	0.002730	
Salience.AInsula (R) (47,14,0)-DefaultMode.LP (L) (−39,−77,33)			−4.01	0.001723	0.012133	
Salience.ACC (0,22,35)—DefaultMode.LP (R) (47,−67,29)			−3.50	0.004397	0.023218	
Language.IFG (R) (54,28,1)-DefaultMode.LP (L) (−39,−77,33)			−3.48	0.004561	0.023609	
Salience.SMG (L) (−60,−39,31)-DefaultMode.PCC (1,−61,38)			−3.28	0.006602	0.031547	
Salience.SMG (L) (−60,−39,31)-DefaultMode.LP (R) (47,−67,29)			−3.25	0.006973	0.032874	
Language.IFG (R) (54,28,1)-DefaultMode.PCC (1,−61,38)			−3.15	0.008328	0.037200	
Salience.AInsula (L) (−44,13,1)-DefaultMode.LP (R) (47,−67,29)			−3.15	0.008384	0.037200	
Salience.AInsula (R) (47,14,0)-DefaultMode.PCC (1,−61,38)			−3.11	0.008997	0.039586	
Salience.AInsula (R) (47,14,0)-DefaultMode.LP (R) (47,−67,29)			−3.10	0.009192	0.040112	
Salience.AInsula (L) (−44,13,1)-DefaultMode.PCC (1,−61,38)			−2.89	0.013555	0.053015	
Salience.AInsula (L) (−44,13,1)-DefaultMode.LP (L) (−39,−77,33)			−2.82	0.015480	0.057826	
Salience.RPFC (L) (−32,45,27)-DefaultMode.LP (R) (47,−67,29)			−2.68	0.020053	0.070014	
DorsalAttention.IPS (R) (39,−42,54)-DefaultMode.PCC (1,−61,38)			−2.50	0.027781	0.090488	
Cluster 5/40						
	Mass	299.41		0.000352	0.002818	0.008000
Language.pSTG (L) (−57,−47,15)-FrontoParietal.PPC (L) (−46,−58,49)			7.03	0.000014	0.000605	
FrontoParietal.PPC (L) (−46,−58,49)-FrontoParietal.LPFC (L) (−43,33,28)			5.82	0.000082	0.001674	
Language.IFG (L) (−51,26,2)-FrontoParietal.LPFC (L) (−43,33,28)			5.32	0.000182	0.002684	
Language.IFG (L) (−51,26,2)-Language.pSTG (L) (−57,−47,15)			5.19	0.000227	0.002910	
Language.pSTG (L) (−57,−47,15)-Cerebellar.Posterior (0,−79,−32)			2.40	0.033319	0.102283	
Language.IFG (L) (−51,26,2)-FrontoParietal.PPC (L) (−46,−58,49)			2.34	0.037644	0.111663	
Cluster 6/40						
	Mass	254.00		0.000515	0.003435	0.011000
Language.pSTG (R) (59,−42,13)-Language.pSTG (L) (−57,−47,15)			5.32	0.000183	0.002684	
FrontoParietal.PPC (R) (52,−52,45)-FrontoParietal.PPC (L) (−46,−58,49)			5.30	0.000188	0.002684	
Grey Matter—FrontoParietal.PPC (L) (−46,−58,49)			5.25	0.000204	0.002730	
Grey Matter—Language.pSTG (L) (−57,−47,15)			4.42	0.000841	0.007526	
Language.pSTG (R) (59,−42,13)-Language.IFG (L) (−51,26,2)			3.09	0.009410	0.040394	
Grey Matter—Language.IFG (L) (−51,26,2)			2.76	0.017323	0.063518	
Grey Matter—SensoriMotor.Lateral (L) (−55,−12,29)			2.53	0.026353	0.086966	
Cluster 7/40						
	Mass	205.24		0.000956	0.005135	0.019000
FrontoParietal.LPFC (R) (41,38,30)-SensoriMotor.Lateral (L) (−55,−12,29)			−4.08	0.001520	0.011147	
FrontoParietal.PPC (R) (52,−52,45)-DorsalAttention.FEF (L) (−27,−9,64)			−3.61	0.003558	0.019984	
Grey Matter—SensoriMotor.Superior (0,−31,67)			−3.58	0.003766	0.020715	
FrontoParietal.PPC (R) (52,−52,45)-SensoriMotor.Lateral (L) (−55,−12,29)			−3.28	0.006632	0.031547	
FrontoParietal.LPFC (R) (41,38,30)-SensoriMotor.Superior (0,−31,67)			−3.19	0.007721	0.035146	
Grey Matter—DorsalAttention.FEF (L) (−27,−9,64)			−3.17	0.008047	0.036313	
Language.pSTG (R) (59,−42,13)-DorsalAttention.IPS (L) (−39,−43,52)			−2.88	0.013836	0.053718	
FrontoParietal.LPFC (R) (41,38,30)-DorsalAttention.FEF (L) (−27,−9,64)			−2.82	0.015552	0.057826	
Language.pSTG (R) (59,−42,13)-DorsalAttention.FEF (L) (−27,−9,64)			−2.60	0.023087	0.079157	
DefaultMode.LP (R) (47,−67,29)-DorsalAttention.FEF (L) (−27,−9,64)			−2.46	0.029823	0.094292	
Cluster 8/40						
	Mass	198.47		0.001027	0.005135	0.020000
Grey Matter—Visual.Lateral (R) (38,−72,13)			5.17	0.000231	0.002910	
Grey Matter—Visual.Medial (2,−79,12)			5.15	0.000241	0.002916	
DefaultMode.LP (L) (−39,−77,33)-Visual.Lateral (L) (−37,−79,10)			3.91	0.002070	0.013662	
DefaultMode.LP (R) (47,−67,29)-Visual.Lateral (R) (38,−72,13)			3.76	0.002729	0.016950	
Grey Matter—Visual.Lateral (L) (−37,−79,10)			3.41	0.005207	0.026435	
Language.pSTG (R) (59,−42,13)-Visual.Lateral (R) (38,−72,13)			2.22	0.046641	0.133840	

**Table A3:** Statistical Values of ROI-to-ROI Connectivity Analysis for Wrestlers.

Analysis Unit			T (12)-Value	p-unc	p-FDR	*p*-FWE
Cluster 1/36						
	Mass	5664.04		0.000000	0.000000	0.000000
DefaultMode.LP (R) (47,−67,29)-DefaultMode.LP (L) (−39,−77,33)			13.38	0.000000	0.000004	
Visual.Lateral (R) (38,−72,13)-Visual.Lateral (L) (−37,−79,10)			12.27	0.000000	0.000007	
Salience.AInsula (R) (47,14,0)-Salience.RPFC (R) (32,46,27)			10.86	0.000000	0.000017	
Language.IFG (R) (54,28,1)-Salience.AInsula (R) (47,14,0)			10.75	0.000000	0.000017	
Cerebellar.Posterior (0,−79,−32)-Cerebellar.Anterior (0,−63,−30)			10.61	0.000000	0.000017	
Language.pSTG (R) (59,−42,13)-Language.IFG (R) (54,28,1)			10.45	0.000000	0.000017	
Salience.SMG (R) (62,−35,32)-Salience.AInsula (R) (47,14,0)			10.10	0.000000	0.000021	
FrontoParietal.LPFC (R) (41,38,30)-FrontoParietal.PPC (R) (52,−52,45)			10.00	0.000000	0.000021	
Visual.Occipital (0,−93,−4)-Cerebellar.Posterior (0,−79,−32)			9.69	0.000001	0.000024	
Salience.SMG (R) (62,−35,32)-Salience.RPFC (R) (32,46,27)			9.59	0.000001	0.000025	
Visual.Lateral (R) (38,−72,13)-Visual.Occipital (0,−93,−4)			9.31	0.000001	0.000031	
Salience.RPFC (R) (32,46,27)-FrontoParietal.LPFC (R) (41,38,30)			8.63	0.000002	0.000053	
Salience.RPFC (R) (32,46,27)-Salience.ACC (0,22,35)			8.51	0.000002	0.000055	
Visual.Medial (2,−79,12)-Visual.Occipital (0,−93,−4)			8.35	0.000002	0.000063	
Salience.ACC (0,22,35)—SensoriMotor.Lateral (R) (56,−10,29)			8.33	0.000002	0.000063	
Visual.Lateral (L) (−37,−79,10)-Visual.Occipital (0,−93,−4)			7.97	0.000004	0.000093	
Visual.Lateral (R) (38,−72,13)-Visual.Medial (2,−79,12)			7.91	0.000004	0.000097	
Visual.Lateral (L) (−37,−79,10)-Visual.Medial (2,−79,12)			7.88	0.000004	0.000097	
Language.pSTG (R) (59,−42,13)-Salience.SMG (R) (62,−35,32)			7.72	0.000005	0.000110	
Grey Matter—Language.pSTG (R) (59,−42,13)			7.04	0.000014	0.000232	
Salience.AInsula (R) (47,14,0)-Salience.ACC (0,22,35)			6.88	0.000017	0.000281	
Language.pSTG (R) (59,−42,13)-Salience.AInsula (R) (47,14,0)			6.85	0.000018	0.000282	
DefaultMode.LP (R) (47,−67,29)-Grey Matter			6.25	0.000043	0.000556	
DefaultMode.LP (L) (−39,−77,33)-Grey Matter			6.02	0.000060	0.000755	
DefaultMode.LP (L) (−39,−77,33)-Visual.Lateral (L) (−37,−79,10)			5.87	0.000076	0.000928	
DefaultMode.LP (R) (47,−67,29)-DefaultMode.MPFC (1,55,−3)			5.86	0.000078	0.000932	
Salience.SMG (R) (62,−35,32)-Salience.ACC (0,22,35)			5.69	0.000101	0.001158	
DefaultMode.LP (R) (47,−67,29)-Language.pSTG (R) (59,−42,13)			5.61	0.000114	0.001282	
DefaultMode.LP (L) (−39,−77,33)-Visual.Lateral (R) (38,−72,13)			5.50	0.000137	0.001473	
Salience.AInsula (R) (47,14,0)-SensoriMotor.Lateral (R) (56,−10,29)			5.47	0.000144	0.001522	
DefaultMode.LP (L) (−39,−77,33)-DefaultMode.MPFC (1,55,−3)			5.40	0.000161	0.001571	
DefaultMode.LP (R) (47,−67,29)-Visual.Lateral (R) (38,−72,13)			5.05	0.000284	0.002481	
Salience.SMG (R) (62,−35,32)-DorsalAttention.IPS (R) (39,−42,54)			4.91	0.000357	0.002899	
Language.IFG (R) (54,28,1)-Salience.SMG (R) (62,−35,32)			4.80	0.000435	0.003427	
DefaultMode.MPFC (1,55,−3)-Visual.Lateral (R) (38,−72,13)			4.70	0.000518	0.003908	
Language.IFG (R) (54,28,1)-Salience.RPFC (R) (32,46,27)			4.60	0.000607	0.004512	
Language.pSTG (R) (59,−42,13)-Salience.RPFC (R) (32,46,27)			4.50	0.000730	0.005075	
DefaultMode.PCC (1,−61,38)-Grey Matter			4.46	0.000785	0.005320	
Salience.RPFC (R) (32,46,27)-FrontoParietal.PPC (R) (52,−52,45)			4.46	0.000786	0.005320	
Grey Matter—SensoriMotor.Lateral (R) (56,−10,29)			4.43	0.000825	0.005340	
Salience.SMG (R) (62,−35,32)-FrontoParietal.PPC (R) (52,−52,45)			4.28	0.001077	0.006614	
Language.pSTG (R) (59,−42,13)-FrontoParietal.PPC (R) (52,−52,45)			4.22	0.001180	0.007000	
DefaultMode.LP (R) (47,−67,29)-DefaultMode.PCC (1,−61,38)			4.15	0.001343	0.007462	
Visual.Lateral (R) (38,−72,13)-Grey Matter			4.07	0.001543	0.008228	
DefaultMode.MPFC (1,55,−3)-Grey Matter			4.06	0.001586	0.008373	
DefaultMode.PCC (1,−61,38)-DefaultMode.MPFC (1,55,−3)			3.99	0.001791	0.009271	
DorsalAttention.IPS (R) (39,−42,54)-DorsalAttention.FEF (R) (30,−6,64)			3.83	0.002401	0.012075	
DefaultMode.PCC (1,−61,38)-DefaultMode.LP (L) (−39,−77,33)			3.78	0.002614	0.012857	
Visual.Occipital (0,−93,−4)-Cerebellar.Anterior (0,−63,−30)			3.78	0.002642	0.012857	
Language.IFG (R) (54,28,1)-FrontoParietal.LPFC (R) (41,38,30)			3.77	0.002678	0.012857	
Visual.Medial (2,−79,12)-Grey Matter			3.73	0.002885	0.013723	
SensoriMotor.Lateral (R) (56,−10,29)-DorsalAttention.IPS (R) (39,−42,54)			3.54	0.004059	0.018120	
FrontoParietal.PPC (R) (52,−52,45)-DorsalAttention.IPS (R) (39,−42,54)			3.54	0.004084	0.018120	
Language.IFG (R) (54,28,1)-FrontoParietal.PPC (R) (52,−52,45)			3.52	0.004190	0.018382	
Grey Matter—Salience.SMG (R) (62,−35,32)			3.41	0.005163	0.020639	
FrontoParietal.LPFC (R) (41,38,30)-SensoriMotor.Lateral (R) (56,−10,29)			3.40	0.005235	0.020639	
DefaultMode.MPFC (1,55,−3)-Language.pSTG (R) (59,−42,13)			3.26	0.006853	0.025131	
Visual.Lateral (L) (−37,−79,10)-Grey Matter			3.25	0.006913	0.025174	
DefaultMode.PCC (1,−61,38)-Language.pSTG (R) (59,−42,13)			3.14	0.008497	0.029711	
DefaultMode.MPFC (1,55,−3)-Visual.Occipital (0,−93,−4)			3.07	0.009674	0.032915	
DefaultMode.LP (L) (−39,−77,33)-Visual.Occipital (0,−93,−4)			3.07	0.009757	0.032915	
Grey Matter—Salience.AInsula (R) (47,14,0)			3.03	0.010401	0.034540	
Visual.Medial (2,−79,12)-Cerebellar.Posterior (0,−79,−32)			2.96	0.011894	0.038766	
Cerebellar.Anterior (0,−63,−30)-Grey Matter			2.94	0.012383	0.039626	
DefaultMode.MPFC (1,55,−3)-Language.IFG (R) (54,28,1)			2.93	0.012498	0.039752	
Salience.AInsula (R) (47,14,0)-DorsalAttention.IPS (R) (39,−42,54)			2.84	0.014905	0.046022	
FrontoParietal.LPFC (R) (41,38,30)-DorsalAttention.FEF (R) (30,−6,64)			2.84	0.015005	0.046062	
Grey Matter—Language.IFG (R) (54,28,1)			2.71	0.019005	0.054834	
Grey Matter—FrontoParietal.PPC (R) (52,−52,45)			2.69	0.019668	0.056440	
FrontoParietal.PPC (R) (52,−52,45)-SensoriMotor.Lateral (R) (56,−10,29)			2.66	0.020889	0.058668	
Salience.AInsula (R) (47,14,0)-FrontoParietal.LPFC (R) (41,38,30)			2.58	0.023945	0.064505	
Salience.SMG (R) (62,−35,32)-SensoriMotor.Lateral (R) (56,−10,29)			2.52	0.027042	0.071751	
Visual.Lateral (R) (38,−72,13)-Cerebellar.Posterior (0,−79,−32)			2.47	0.029499	0.076351	
FrontoParietal.LPFC (R) (41,38,30)-DorsalAttention.IPS (R) (39,−42,54)			2.39	0.034250	0.085706	
Visual.Lateral (R) (38,−72,13)-Language.pSTG (R) (59,−42,13)			2.32	0.038735	0.094909	
DefaultMode.LP (R) (47,−67,29)-Language.IFG (R) (54,28,1)			2.27	0.042268	0.100984	
DefaultMode.MPFC (1,55,−3)-Visual.Lateral (L) (−37,−79,10)			2.24	0.044879	0.106261	
Language.IFG (R) (54,28,1)-SensoriMotor.Lateral (R) (56,−10,29)			2.22	0.046358	0.109272	
Cluster 2/36						
	Mass	1837.04		0.000000	0.000000	0.000000
Salience.SMG (L) (−60,−39,31)-Language.pSTG (L) (−57,−47,15)			13.37	0.000000	0.000004	
Language.pSTG (L) (−57,−47,15)-Language.IFG (L) (−51,26,2)			9.77	0.000000	0.000024	
Salience.AInsula (L) (−44,13,1)-Language.IFG (L) (−51,26,2)			8.80	0.000001	0.000046	
FrontoParietal.LPFC (L) (−43,33,28)-FrontoParietal.PPC (L) (−46,−58,49)			7.72	0.000005	0.000110	
Salience.AInsula (L) (−44,13,1)-Salience.SMG (L) (−60,−39,31)			7.18	0.000011	0.000211	
Salience.RPFC (L) (−32,45,27)-Salience.AInsula (L) (−44,13,1)			7.04	0.000014	0.000232	
Salience.AInsula (L) (−44,13,1)-Language.pSTG (L) (−57,−47,15)			6.48	0.000030	0.000430	
FrontoParietal.LPFC (L) (−43,33,28)-Salience.RPFC (L) (−32,45,27)			6.24	0.000043	0.000556	
FrontoParietal.LPFC (L) (−43,33,28)-Language.IFG (L) (−51,26,2)			5.42	0.000155	0.001564	
Salience.SMG (L) (−60,−39,31)-Language.IFG (L) (−51,26,2)			5.24	0.000207	0.001914	
DorsalAttention.IPS (L) (−39,−43,52)-Salience.SMG (L) (−60,−39,31)			5.05	0.000287	0.002481	
FrontoParietal.PPC (L) (−46,−58,49)-Language.pSTG (L) (−57,−47,15)			4.73	0.000488	0.003791	
FrontoParietal.PPC (L) (−46,−58,49)-Language.IFG (L) (−51,26,2)			4.55	0.000670	0.004783	
FrontoParietal.LPFC (L) (−43,33,28)-SensoriMotor.Lateral (L) (−55,−12,29)			4.42	0.000829	0.005340	
DorsalAttention.IPS (L) (−39,−43,52)-FrontoParietal.LPFC (L) (−43,33,28)			4.28	0.001077	0.006614	
Salience.RPFC (L) (−32,45,27)-Language.pSTG (L) (−57,−47,15)			4.22	0.001187	0.007000	
SensoriMotor.Lateral (L) (−55,−12,29)-Salience.AInsula (L) (−44,13,1)			4.14	0.001378	0.007578	
Salience.RPFC (L) (−32,45,27)-Salience.SMG (L) (−60,−39,31)			4.10	0.001460	0.007887	
Salience.RPFC (L) (−32,45,27)-Language.IFG (L) (−51,26,2)			3.95	0.001926	0.009779	
DorsalAttention.IPS (L) (−39,−43,52)-Salience.AInsula (L) (−44,13,1)			3.41	0.005168	0.020639	
DorsalAttention.IPS (L) (−39,−43,52)-Language.pSTG (L) (−57,−47,15)			3.34	0.005848	0.022056	
DorsalAttention.FEF (L) (−27,−9,64)-Language.IFG (L) (−51,26,2)			3.09	0.009312	0.031926	
FrontoParietal.PPC (L) (−46,−58,49)-Salience.SMG (L) (−60,−39,31)			3.07	0.009787	0.032915	
FrontoParietal.LPFC (L) (−43,33,28)-Salience.AInsula (L) (−44,13,1)			2.95	0.012192	0.039253	
SensoriMotor.Lateral (L) (−55,−12,29)-Salience.SMG (L) (−60,−39,31)			2.74	0.018032	0.053390	
DorsalAttention.IPS (L) (−39,−43,52)-Salience.RPFC (L) (−32,45,27)			2.66	0.020841	0.058668	
DorsalAttention.IPS (L) (−39,−43,52)-SensoriMotor.Lateral (L) (−55,−12,29)			2.63	0.022131	0.061247	
DorsalAttention.IPS (L) (−39,−43,52)-FrontoParietal.PPC (L) (−46,−58,49)			2.44	0.031052	0.079589	
FrontoParietal.LPFC (L) (−43,33,28)-Language.pSTG (L) (−57,−47,15)			2.39	0.034182	0.085706	
SensoriMotor.Lateral (L) (−55,−12,29)-Language.pSTG (L) (−57,−47,15)			2.38	0.034450	0.085801	
SensoriMotor.Lateral (L) (−55,−12,29)-Language.IFG (L) (−51,26,2)			2.31	0.039670	0.096080	
Cluster 3/36						
	Mass	1040.56		0.000000	0.000000	0.000000
Salience.AInsula (R) (47,14,0)-Salience.AInsula (L) (−44,13,1)			9.06	0.000001	0.000039	
Salience.RPFC (R) (32,46,27)- Salience.RPFC (L) (−32,45,27)			7.10	0.000012	0.000227	
Salience.AInsula (R) (47,14,0)-Salience.RPFC (L) (−32,45,27)			6.51	0.000029	0.000424	
Grey Matter –Language.pSTG (L) (−57,−47,15)			6.38	0.000035	0.000485	
Salience.ACC (0,22,35)—Salience.RPFC (L) (−32,45,27)			5.41	0.000157	0.001564	
Salience.ACC (0,22,35)—Salience.AInsula (L) (−44,13,1)			5.22	0.000216	0.001964	
Salience.AInsula (R) (47,14,0)-Salience.SMG (L) (−60,−39,31)			5.12	0.000255	0.002282	
Language.pSTG (R) (59,−42,13)-Language.pSTG (L) (−57,−47,15)			5.01	0.000302	0.002569	
Salience.SMG (R) (62,−35,32)-Salience.SMG (L) (−60,−39,31)			5.00	0.000311	0.002569	
Language.IFG (R) (54,28,1)-Salience.AInsula (L) (−44,13,1)			4.36	0.000931	0.005850	
Salience.SMG (R) (62,−35,32)-Salience.AInsula (L) (−44,13,1)			4.22	0.001199	0.007000	
Salience.SMG (R) (62,−35,32)-Salience.RPFC (L) (−32,45,27)			4.20	0.001239	0.007110	
Language.IFG (R) (54,28,1)-Language.IFG (L) (−51,26,2)			3.98	0.001811	0.009282	
Language.IFG (R) (54,28,1)-Salience.SMG (L) (−60,−39,31)			3.48	0.004551	0.019695	
Salience.RPFC (R) (32,46,27)-Salience.AInsula (L) (−44,13,1)			3.46	0.004703	0.020067	
Grey Matter—Salience.SMG (L) (−60,−39,31)			3.40	0.005228	0.020639	
Language.IFG (R) (54,28,1)-Language.pSTG (L) (−57,−47,15)			2.77	0.016927	0.051073	
Salience.RPFC (R) (32,46,27)-Salience.SMG (L) (−60,−39,31)			2.77	0.017074	0.051223	
Grey Matter—Language.IFG (L) (−51,26,2)			2.73	0.018100	0.053390	
Salience.SMG (R) (62,−35,32)-Language.pSTG (L) (−57,−47,15)			2.66	0.020775	0.058668	
Cerebellar.Anterior (0,−63,−30)-Salience.SMG (L) (−60,−39,31)			2.58	0.023878	0.064505	
Salience.AInsula (R) (47,14,0)-Language.IFG (L) (−51,26,2)			2.57	0.024527	0.065404	
Salience.AInsula (R) (47,14,0)-Language.pSTG (L) (−57,−47,15)			2.50	0.027914	0.073327	
Language.pSTG (R) (59,−42,13)-Salience.SMG (L) (−60,−39,31)			2.41	0.032858	0.083408	
Grey Matter—Salience.AInsula (L) (−44,13,1)			2.33	0.038176	0.094192	
Salience.AInsula (R) (47,14,0)-SensoriMotor.Lateral (L) (−55,−12,29)			2.27	0.042137	0.100984	
Cluster 4/36						
	Mass	668.06		0.000000	0.000000	0.000000
DefaultMode.LP (R) (47,−67,29)-Salience.RPFC (L) (−32,45,27)			−8.54	0.000002	0.000055	
Visual.Lateral (R) (38,−72,13)-FrontoParietal.LPFC (L) (−43,33,28)			−7.18	0.000011	0.000211	
Visual.Lateral (R) (38,−72,13)-Salience.RPFC (L) (−32,45,27)			−6.70	0.000022	0.000339	
Visual.Lateral (R) (38,−72,13)-SensoriMotor.Lateral (L) (−55,−12,29)			−5.56	0.000123	0.001356	
DefaultMode.LP (R) (47,−67,29)-SensoriMotor.Lateral (L) (−55,−12,29)			−5.36	0.000171	0.001639	
DefaultMode.PCC (1,−61,38)-SensoriMotor.Lateral (L) (−55,−12,29)			−4.21	0.001206	0.007000	
DefaultMode.PCC (1,−61,38)-Salience.AInsula (L) (−44,13,1)			−3.61	0.003594	0.016358	
DefaultMode.LP (R) (47,−67,29)-Salience.AInsula (L) (−44,13,1)			−3.40	0.005230	0.020639	
DefaultMode.MPFC (1,55,−3)-DorsalAttention.IPS (L) (−39,−43,52)			−3.37	0.005605	0.021292	
DefaultMode.MPFC (1,55,−3)-Salience.RPFC (L) (−32,45,27)			−3.26	0.006854	0.025131	
DefaultMode.LP (L) (−39,−77,33)-Salience.RPFC (L) (−32,45,27)			−2.89	0.013679	0.042991	
DefaultMode.LP (R) (47,−67,29)-Salience.SMG (L) (−60,−39,31)			−2.84	0.014817	0.046020	
Visual.Lateral (R) (38,−72,13)-FrontoParietal.PPC (L) (−46,−58,49)			−2.64	0.021398	0.059779	
DefaultMode.MPFC (1,55,−3)-SensoriMotor.Lateral (L) (−55,−12,29)			−2.63	0.022156	0.061247	
Visual.Lateral (R) (38,−72,13)-DorsalAttention.IPS (L) (−39,−43,52)			−2.32	0.039048	0.095011	
Visual.Lateral (R) (38,−72,13)-DorsalAttention.FEF (L) (−27,−9,64)			−2.25	0.044029	0.104718	
Cluster 5/36						
	Mass	223.06		0.000255	0.001838	0.007000
SensoriMotor.Lateral (R) (56,−10,29)-SensoriMotor.Lateral (L) (−55,−12,29)			8.88	0.000001	0.000045	
FrontoParietal.PPC (R) (52,−52,45)-FrontoParietal.PPC (L) (−46,−58,49)			5.72	0.000096	0.001127	
Cluster 6/36						
	Mass	184.98		0.000662	0.003971	0.017000
Visual.Lateral (L) (−37,−79,10)-FrontoParietal.PPC (R) (52,−52,45)			−4.38	0.000898	0.005711	
Visual.Lateral (L) (−37,−79,10)-Salience.ACC (0,22,35)			−4.23	0.001173	0.007000	
Visual.Lateral (L) (−37,−79,10)-SensoriMotor.Lateral (R) (56,−10,29)			−3.78	0.002625	0.012857	
Visual.Lateral (L) (−37,−79,10)-Salience.RPFC (R) (32,46,27)			−3.69	0.003084	0.014411	
Visual.Lateral (L) (−37,−79,10)-FrontoParietal.LPFC (R) (41,38,30)			−3.44	0.004880	0.020450	
Visual.Lateral (R) (38,−72,13)-Salience.ACC (0,22,35)			−3.15	0.008334	0.029486	
Visual.Lateral (R) (38,−72,13)-Salience.RPFC (R) (32,46,27)			−2.40	0.033697	0.085130	
Cluster 7/36						
	Mass	163.64		0.001003	0.005158	0.027000
FrontoParietal.LPFC (R) (41,38,30)-SensoriMotor.Superior (0,−31,67)			−4.72	0.000497	0.003800	
FrontoParietal.LPFC (R) (41,38,30)-DorsalAttention.IPS (L) (−39,−43,52)			−4.43	0.000817	0.005340	
FrontoParietal.LPFC (R) (41,38,30)-DorsalAttention.FEF (L) (−27,−9,64)			−3.40	0.005316	0.020639	
FrontoParietal.PPC (R) (52,−52,45)-DorsalAttention.FEF (L) (−27,−9,64)			−2.83	0.015138	0.046203	
FrontoParietal.PPC (R) (52,−52,45)-FrontoParietal.LPFC (L) (−43,33,28)			−2.81	0.015800	0.047946	
FrontoParietal.PPC (R) (52,−52,45)-SensoriMotor.Superior (0,−31,67)			−2.66	0.020742	0.058668	
SensoriMotor.Lateral (R) (56,−10,29)-FrontoParietal.LPFC (L) (−43,33,28)			−2.32	0.038826	0.094909	
Cluster 8/36						
	Mass	139.18		0.001634	0.007355	0.039000
Salience.RPFC (R) (32,46,27)-SensoriMotor.Superior (0,−31,67)			−6.34	0.000037	0.000504	
Language.IFG (R) (54,28,1)-SensoriMotor.Superior (0,−31,67)			−3.43	0.004953	0.020593	
Salience.AInsula (R) (47,14,0)-SensoriMotor.Superior (0,−31,67)			−3.20	0.007703	0.027858	
Salience.SMG (R) (62,−35,32)-SensoriMotor.Superior (0,−31,67)			−2.72	0.018543	0.053796	
